# Comparative pangenomics of Streptococcus pneumoniae from Malawi: uncovering genetic variability and pathogenicity

**DOI:** 10.1099/mgen.0.001370

**Published:** 2025-04-15

**Authors:** Arash Iranzadeh, Arghavan Alisoltani, Anmol M. Kiran, Robert F. Breiman, Chrispin Chaguza, Chikondi Peno, Jennifer E. Cornick, Dean B. Everett, Nicola Mulder

**Affiliations:** 1 Computational Biology Division, Department of Integrative Biomedical Sciences, Institute of Infectious Disease and Molecular Medicine, Faculty of Health Sciences, University of Cape Town, Western Cape, South Africa; 2 Department of Microbiology-Immunology, Northwestern University Feinberg School of Medicine, Chicago, Illinois, USA; 3 Department of Medicine, Division of Infectious Diseases, Northwestern University Feinberg School of Medicine, Chicago, Illinois, USA; 4 Center for Pathogen Genomics and Microbial Evolution, Havey Institute for Global Health, Northwestern University Feinberg School of Medicine, Chicago, Illinois, USA; 5 Malawi-Liverpool-Wellcome Trust, Queen Elizabeth Central Hospital, College of Medicine, Blantyre, Malawi; 6 Centre for Inflammation Research, Queens Research Institute, University of Edinburgh, Edinburgh, UK; 7 Rollins School of Public Health, Emory University, Atlanta, Georgia, USA; 8 Infectious Diseases and Oncology Research Institute, University of the Witwatersrand, Johannesburg, South Africa; 9 Parasites and Microbes Programme, Wellcome Sanger Institute, Wellcome Genome Campus, Hinxton, Cambridge, UK; 10 Department of Epidemiology of Microbial Diseases, Yale School of Public Health, Yale University, New Haven, Connecticut, USA; 11 Yale Institute for Global Health, Yale University, New Haven, Connecticut, USA; 12 Institute of Infection, Veterinary and Ecological Sciences, University of Liverpool, Liverpool, UK; 13 Department of Public Health and Epidemiology, College of Medicine and Health Sciences, Khalifa University, Abu Dhabi, UAE; 14 Infection Research Unit, Khalifa University, Abu Dhabi, UAE

**Keywords:** F-type ATP synthase, pangenomics, serotype 1, serotype 5, serotype 12F, *Streptococcus pneumoniae*, V-type ATP synthase

## Abstract

*Streptococcus pneumoniae* is a significant cause of bacterial infections, including pneumonia, meningitis and septicemia, primarily affecting children, the elderly and immunocompromised individuals. This study aimed to elucidate the serotype and lineage distribution and molecular mechanisms underlying pneumococcal invasiveness through a comprehensive pangenomic analysis of 1416 isolates from Malawi. Our analysis comprised 810 isolates from asymptomatic carriers and 606 isolates from patients with bacteraemia or meningitis. We identified 58 serotypes, with serotypes 1, 5 and 12F exhibiting significantly higher prevalence among patients. These serotypes likely exhibit reduced nasopharyngeal colonization and demonstrate rapid dissemination to sterile sites. Notably, these serotypes form a distinct lineage with distinct genomic characteristics, including the absence of V-type ATP synthase. The pangenome analysis revealed two highly conserved surface protein complexes, F-type ATP synthase and SecA1-SecY, which deserve further investigation as potential targets for novel therapeutic interventions.

**Impact Statement**

*Streptococcus pneumoniae* remains a significant health burden in Malawi, despite the introduction of pneumococcal vaccines. While some strains harmlessly colonize the nasopharynx, others rapidly invade sterile sites, causing serious infections. Our study analysed the genetic makeup of a large number of pneumococcal strains from Malawi (1416 isolates), focusing on serotype distribution, lineage and the pangenome structure. We identified serotypes 1, 5 and 12F as highly invasive, exhibiting distinct genetic characteristics, including the absence of V-type ATP synthase. These serotypes were found to belong to a unique lineage and exhibited heightened invasiveness with a shortened nasopharyngeal colonization period, which may facilitate rapid infection. Our findings suggest that these serotypes may possess specific mechanisms contributing to their ability to cause serious infections and reduce colonization, which could be important for the development of more effective prevention and treatment strategies. Additionally, we identified the F-type ATP synthase and SecA1-SecY complexes as conserved surface proteins. While our results are based on computational analysis, they warrant further experimental validation to assess their biological significance and clinical applicability, with the potential to inform future vaccine development and drug discovery efforts.

## Data Summary

All data generated or analysed during this study are publicly available. A total of 1416 raw sequencing reads (FASTQ files) have been deposited in the European Nucleotide Archive (ENA). The sample accession numbers for each sequence, along with the corresponding metadata, are provided in the supplementary Excel file, ‘Data_summary.xlsx’.

## Introduction

*Streptococcus pneumoniae*, also known as pneumococcus, is a Gram-positive, facultatively anaerobic bacterium that can cause invasive pneumococcal disease that contributes to global morbidity and mortality. While the introduction of pneumococcal conjugate vaccine (PCV) has led to a reduction in the incidence of pneumococcal diseases in many countries, the mortality rate associated with pneumococcal infection remains high. According to the World Health Organization, it is estimated that about one million children die from pneumococcal disease every year [1]. Even in the post-PCV era, a substantial disease burden persists in low-income countries, particularly in Africa, including Malawi [2].

Although pneumococcal nasopharyngeal colonization is asymptomatic, it is essential for transmission and the development of disease [3]. Symptoms appear when isolates from the nasopharynx spread to normally sterile sites such as the ears, lungs, blood and central nervous system. Depending on the infected organ, *S. pneumoniae* can cause two types of infection: (i) non-invasive (mucosal) pneumococcal diseases, such as otitis media (ear infection), and (ii) invasive pneumococcal diseases (IPDs) such as pneumonia (lung infection), bacteraemia (blood infection) and meningitis (central nervous system infection). IPD incidence is the highest among infants, the elderly and immunocompromised individuals, most likely due to their less efficient immune systems [4].

Pneumococci possess several virulence factors, including proteins and the polysaccharide capsule [5,6]. The proteins include (i) hydrolases, such as neuraminidase, which aids in bacterial adherence and autolysin, which triggers inflammation; (ii) proteases that degrade host proteins and manipulate immune response; (iii) antigens, such as pneumococcal surface protein A, which stimulates the immune system leading to inflammation; and (iv) hyaluronidase, which helps pneumococci spread through tissues by breaking down substances that hold cells together [7]. The polysaccharide capsule is the most critical pneumococcal virulence factor, enabling pneumococci to evade immune detection during colonization and invasion [8]. Its biosynthesis is regulated by a cluster of genes in the capsular polysaccharide (cps) locus [9]. The cps locus contains genes that encode enzymes responsible for synthesizing and assembling polysaccharides to form a capsule structure around the bacterial cell. Pneumococcal serotypes are defined by variations in the types and arrangements of saccharides in the capsule structure, which arise from variations in the cps locus, specifically through (i) gene variations, where each serotype has a unique set of genes in the cps locus; (ii) the regulation of gene expression, where promoters and phase variation control capsule production levels; and (iii) horizontal gene transfer and recombination, allowing pneumococci to acquire or reshuffle capsule genes from other strains, generating new serotypes. To date, one hundred pneumococcal serotypes have been identified [10]. The immunogenic properties of the capsular polysaccharide have been utilized to develop all pneumococcal vaccines currently in use, including PCV7, PCV10, PCV13 and PCV20, which cover 7, 10, 13 and 20 serotypes, respectively. PCV13 includes the following serotypes: 1, 3, 4, 5, 6A, 6B, 7F, 9V, 14, 18C, 19A, 19F and 23F. PCV20 includes the following additional serotypes: 8, 10A, 11A, 12F, 15B, 22F and 33F. Although the introduction of PCVs has significantly reduced the burden of disease caused by vaccine-type strains, serotype replacement has led to an increase in the carriage rate and incidence of IPDs caused by non-vaccine-type strains [11,12].

In November 2011, PCV13 was introduced in Malawi, which markedly decreased the health system burden and rates of severe childhood pneumonia [13]. Three years after PCV13 introduction in Karonga District in Malawi, vaccine-type pneumococcal carriage decreased in vaccinated children and unvaccinated older individuals, indicating herd protection. However, pneumococcal carriage remained high in infants too young to be vaccinated, and non-vaccine-type carriage increased in vaccinated children, suggesting serotype replacement [14]. Seven years after introducing PCV13, vaccine-eligible children experienced a significant reduction in vaccine-type invasive pneumococcal disease, but indirect protection for unvaccinated infants and adults was limited. Although the overall incidence of the disease has decreased, cases caused by non-vaccine serotypes have increased, highlighting the need for additional vaccination strategies to protect vulnerable populations [15]. Another concern was the emergence of antibiotic-resistant pneumococci due to the overuse of antibiotics in the twenty-first century [16]. To develop new, more effective vaccines against the vaccine-escape clones and design effective drugs against antibiotic-resistant strains, it is critical to understand the functions of genes involved in pneumococcal colonization and pathogenesis. During the past decade, the evolution of high-throughput sequencing technologies has generated enormous amounts of genomic data that have enabled researchers to perform large-scale genomic analysis. A well-known example is pangenome studies. In bacteria, the pangenome refers to the complete set of genes within a particular collection of strains from one or closely related species [17]. It includes the core genome, which consists of genes shared by all strains or the majority of them (> 95%) within the species. Core genes are essential for basic cellular functions and survival. These conserved genes can be considered as potential targets for new vaccines and medications. The accessory genome, on the other hand, includes genes present in some but not all strains, contributing to diversity in traits such as pathogenicity, antibiotic resistance and adaptation to different environments. The pangenome is useful for analysing genetically diverse and recombinogenic pathogens such as *S. pneumoniae*.

Determining the invasive pneumococcal strains and their genetic structure is a promising area of research that can provide valuable insights into the prevention and treatment of pneumococcal disease, ultimately reducing mortality rates. In this study, we conducted whole-genome sequencing on 1416 pneumococcal isolates from residents of Blantyre, Karonga, and Lilongwe in Malawi. Our study aims to (i) identify the distribution of pneumococcal serotypes and lineages in Malawi, (ii) identify the most invasive serotypes and lineages, (iii) characterize the pneumococcal pangenome structure and its relationship to invasive strains and (iv) identify conserved genes, their functions and gene variants in invasive strains.

## Methods

### Study design and sample collection

The study utilized archived samples maintained by the Malawi-Liverpool Wellcome Trust Clinical Research Programme (MLW). Samples were collected from individuals residing in three distinct regions of Malawi: Blantyre in the south, Karonga in the north and Lilongwe in the central region. This cohort included isolates from both healthy carriers and symptomatic patients.

Carriage samples were collected from the nasopharynx of healthy individuals as part of the Health and Demographic Surveillance System in Karonga and Blantyre between 2009 and 2014, using nasopharyngeal swabs. Pneumococcal isolates were identified following a previously established protocol [18]. Briefly, isolates were cultured on blood agar supplemented with gentamicin, and their identity was confirmed through optochin disc assays, colony morphology, alpha-hemolysis and optochin susceptibility, in accordance with standard pneumococcal identification protocols. To capture the diversity of carriage, only a single colony from each sample was sequenced, ensuring that no sample contained multiple serotypes.

Invasive pneumococcal isolates were obtained from archived bacterial samples at MLW, collected from blood or cerebrospinal fluid (CSF) of symptomatic patients attending Queen Elizabeth Central Hospital in Blantyre and Kamuzu Central Hospital in Lilongwe between 1997 and 2015. The selection of these isolates was blinded to their serotypes, ensuring an unbiased representation of serotype prevalence in the disease group. Isolates were then streaked onto blood agar plates supplemented with gentamicin, and optochin tests were performed as described in reference [19].

This study did not include paired samples. Each participant contributed only one sample during data collection: either a nasopharyngeal swab from healthy individuals or a blood or CSF sample from symptomatic patients. In this context, ‘sterile sites’ refers specifically to blood and CSF.

### Whole-genome sequencing and quality control

Archived carriage and invasive isolates were sequenced as part of the Global Pneumococcal Sequencing Project and the Pneumococcal African Genomic Consortium at the Wellcome Trust Sanger Institute (UK), with funding from the Bill and Melinda Gates Foundation (grant number: 079828). Bacterial DNA was extracted using the QIAamp DNA Mini Kit and the QIAgen BioRobot (QIAGEN). Sequencing was performed on Illumina Genome Analyzer II and HiSeq platforms, generating 125 bp paired-end reads. The quality of read was assessed using FastQC [20] and MultiQC [21].

### Genome assembly and annotation

Genomes were assembled using VelvetOptimiser [22], a tool designed to optimize the Velvet genome assembler by adjusting parameters for improved contig assembly. Settings were configured to generate contigs longer than 500 base pairs, with a hash range set from 61 to 119 to balance assembly sensitivity and accuracy. The quality of the assembled genomes was assessed using QUAST [23], which provides comprehensive evaluation metrics for assembly quality, including contiguity, accuracy and completeness. To ensure the inclusion of only high-quality assemblies, filtering thresholds were applied. Assemblies with contigs shorter than 500 base pairs were excluded, as they were deemed too fragmented. Additionally, assemblies with an N50 value lower than 1000 base pairs were discarded to avoid unreliable, fragmented genomes. Finally, only assemblies with greater than 90% completeness were retained for further analysis. Genome annotation was performed using Prokka [24], a software tool that automates the annotation of bacterial genomes by predicting gene features and functional annotations based on a comprehensive set of databases.

### *In silico* serotyping, lineage assignment and sequence typing

SeroBA [25] was used to perform *in silico* serotyping of the isolates by inferring the pneumococcal serotype directly from the raw paired-end sequencing reads. This tool employs a *k*-mer-based approach, which identifies unique *k*-mer sequences associated with different serotypes, allowing for accurate serotype determination.

The PopPUNK tool [26] was employed to cluster isolates into lineages known as Global Pneumococcal Sequence Clusters (GPSCs). PopPUNK categorizes bacterial genomes into distinct GPSCs by analysing genome-wide distances derived from sequence *k*-mer content. For *S. pneumoniae*, we used version 4 of the pre-built database provided by PopPUNK, which includes 42 157 isolates and incorporates GPSCs for compatibility with established nomenclature systems (https://www.bacpop.org/poppunk/). This approach provides detailed insights into the genetic diversity of pneumococcal populations while ensuring consistency with globally recognized cluster schemes.

To determine the sequence type (ST) of the pneumococcal isolates, we applied the multilocus sequence typing (MLST) approach using the Short Read Sequence Typing 2 (SRST2) tool [27]. SRST2 allows for the accurate determination of STs directly from raw sequencing reads. This tool maps reads to the reference alleles of seven housekeeping genes (aroE, gdh, gki, recP, spi, xpt and ddl) used in the MLST scheme for *S. pneumoniae*. SRST2 identified the allelic profile of each isolate by comparing the gene sequences to those in the PubMLST database [27], assigning a specific ST to each unique combination of alleles across the seven loci. To confirm the accuracy of serotype, GPSC and ST assignments, the isolates were separately submitted to the Pathogenwatch web platform [28].

### Statistical analysis of serotype distribution

Any serotype, GPSC and ST with a relative frequency greater than 5% in either collection source (nasopharynx, blood or cerebrospinal fluid) was classified as abundant. Fisher’s exact test was applied to each serotype to assess whether its presence in the carriage group differed significantly from that in the patient group. The Benjamini–Hochberg method was used to adjust *P*-values, controlling the false discovery rate, with a significance threshold set at 0.01. The odds ratio (OR) for serotype *k* was calculated using the formula: *OR = (ad)/(bc*), where *a* represents the number of invasive serotype *k*, *b* is the number of carriage serotype *k*, *c* is the number of invasive non-serotype *k* and *d* is the number of carriage non-serotype *k*.

### Pangenome construction

To construct the pangenome of the pneumococcal collection, Panaroo [29], which applies a graph-based algorithm, was deployed. Panaroo utilizes information from all genomes within the dataset to improve the clustering of paralogous genes, resulting in a comprehensive representation of the pangenome. To ensure data quality and identify potential issues with the annotated samples before running the pan-genome analysis, we first applied the Panaroo pre-processing script (panaroo-qc). This script incorporates the widely used Mash algorithm and generates plots to detect potential sources of contamination and outlier samples. Samples flagged for contamination were excluded from subsequent analyses. After data cleaning, Panaroo was run with its default settings to classify genes within the pan-genome based on their presence–absence patterns across the analysed strains. Core genes are the most conserved, identified in at least 99–100% of the isolates. Soft core genes exhibit high prevalence but slightly less conservation, being present in 95–99% of isolates. Shell genes are found in 15–95% of isolates, while cloud genes, representing the most variable genes, are identified in less than 15% of isolates. The core genome consists of core and soft core genes, while the accessory genome includes shell and cloud genes. Panaroo generates several output files for downstream analyses, including a core gene alignment and a gene presence–absence matrix. This matrix provides a detailed record of the presence or absence of each gene cluster across the isolates, facilitating integration with subsequent analyses such as phylogenetics, population structure, and gene presence–absence investigations.

### Characterizing population structure

Population structure was determined using the phylogenetic analysis and principal component analysis (PCA). The phylogenetic tree highlights variation in the core genome, evidenced by small-scale variants, including SNPs and insertions and deletions (Indels). In contrast, PCA of gene distribution reveals variation in the accessory genome, evidenced by large-scale variants in the form of whole gene presence–absence.

A phylogenetic tree was constructed using the core gene alignment generated by Panaroo. The alignment was analysed with IQ-TREE 2 [30] to infer the tree using the maximum likelihood algorithm and the general time-reversible substitution model, which accommodates different rates of substitution between all nucleotide pairs. The resulting phylogenetic tree was visualized using the R packages ggplot2 [31] and ggtree [32], which enabled the integration of serotypes. Dimensionality reduction was performed using PCA on the gene presence–absence matrix after removing columns with zero variance, using the MixOmics package in R [33]. This step reduced the complexity of the data while preserving the variance. The 3D PCA plot was created using the plotly package in R [34] to provide an interactive visualization, allowing a more comprehensive exploration of the population structure.

### Gene presence–absence and functional enrichment analysis

The gene presence–absence statistical analysis was conducted using Scoary [35], which evaluates the association between genes in the pangenome and phenotypic traits while accounting for population stratification to minimize bias. The analysis was performed across isolates with different phenotypic traits to characterize the pangenome structure and its potential association with virulence. Phenotypic traits were assigned to isolates based on their invasiveness and population structure (see the ‘Results’ section). A Bonferroni correction was applied to adjust for multiple testing, and genes with a corrected *P*-value of less than 0.01 were considered statistically significant.

It is worth noting that identifying homologous genes across the core and accessory genomes is likely in *S. pneumoniae*. Due to frequent genetic recombination and horizontal gene transfer, *S. pneumoniae* often harbours genes with similar functions in both the core and accessory genomes. Before performing gene mapping and functional enrichment analysis, we conducted a filtering step to ensure that significant gene functions were unique to the accessory genome. This involved excluding genes with paralogs in the core genome by performing a sequence-based comparison. Using blast [36], we aligned all significant genes against the core genome to detect and remove homologous sequences. This filtering step ensured that only accessory genes with no similar function in the core genome were retained in the final list of significant genes. The same approach was applied to identify homologous genes between the sets of genes present and absent in a given trait, excluding them from the functional analysis.

To identify potential functional relationships among the significant genes, a functional enrichment analysis was conducted using STRING [37], which predicts both direct (physical) and indirect (functional) protein–protein interactions by integrating data from various sources, including genomic context, lab experiments, co-expression, automated text mining and curated databases. STRING employs classification systems such as Kyoto Encyclopedia of Genes and Genomes (KEGG) [38] and Pfam [39]. To perform the analysis, the complete proteome of *S. pneumoniae* TIGR4 (GenBank: AE005672.3) was downloaded from the National Center for Biotechnology Information (NCBI) database and manually integrated into STRING using the ‘Add Organism’ feature. TIGR4, a well-characterized, pathogenic serotype 4 strain, was chosen as it provides broad gene content coverage, making it suitable for studying virulence factors across diverse pneumococcal strains. Using the ‘Multiple Sequences’ tool in STRING, TIGR4 was selected as the reference organism. Enriched pathways with a false discovery rate of less than 0.01 were identified, providing insights into the biological pathways, interactions and functional roles associated with the significant genes. To identify the locations of core and significant accessory genes on the genome, the selected reference genome was uploaded to the Proksee [40] using GenBank accession number: AE005672.3. The locations of genes on the chromosome were then identified using the ‘Features’ tool.

## Results

A total of 1480 isolates were sequenced, comprising 826 isolates from the nasopharynx of healthy carriers, 369 isolates from the blood of bacteraemia patients, and 285 isolates from the CSF of meningitis patients. Of these, 1416 samples passed quality control and were included in the analysis. The demographics of the samples are summarized in Table 1.

**Table 1.** Demographics of 1416 pneumococcal isolates collected from Malawi (quality control passed)

Characteristics	Categories	Nasopharynx (*n*=810)	Blood (*n*=345)	CSF (*n*=261)
**Age (in years)**	**< 5**	535 (66%)	158 (46%)	134 (51%)
	**5–19**	106 (13%)	38 (11%)	55 (21%)
	**20–40**	57 (7%)	63 (18%)	45 (17%)
	**> 40**	7 (1%)	20 (6%)	7 (3%)
	**Unknown**	105 (13%)	66 (19%)	20 (8%)
**Sex**	**Female**	396 (49%)	123 (36%)	103 (39%)
	**Male**	308 (38%)	114 (33%)	114 (44%)
	**Unknown**	106 (13%)	108 (31%)	44 (17%)
**City**	**Blantyre**	168 (21%)	334 (97%)	238 (91%)
	**Karonga**	642 (79%)	0	0
	**Lilongwe**	0	0	22 (8%)
	**Unknown**	0	11 (3%)	1 (1%)
**Sampling period**		2009–2015	1997–2015	2000–2015

### Serotypes, GPSCs and ST distribution

**Fig. 1.**
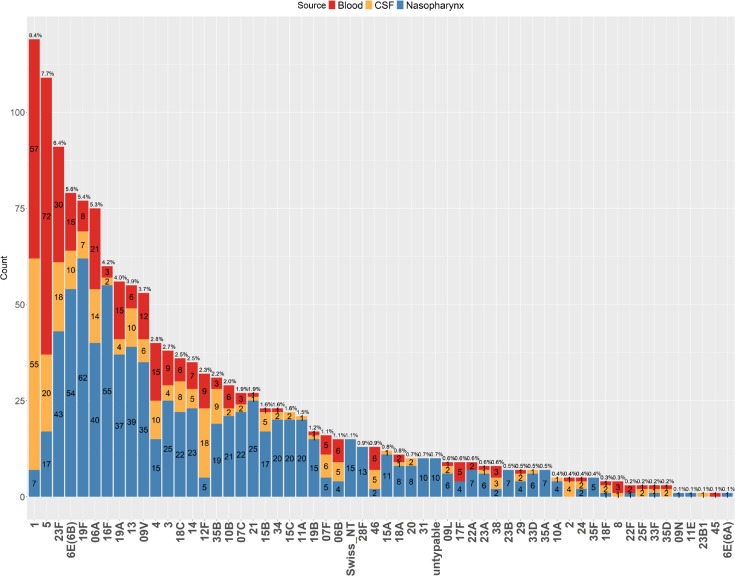
Distribution of pneumococcal serotypes in 1416 Malawi isolates. The distribution of pneumococcal serotypes across nasopharyngeal, blood and CSF isolates collected in Malawi. Each bar represents the number of isolates positive for a particular serotype within a given collection source. Serotypes are arranged in descending order based on their relative frequency in the dataset indicated above each bar.

Altogether, the isolates represented 58 distinct serotypes. Regardless of the collection source (nasopharynx, blood and CSF), serotypes 1 (8.4%), 5 (7.7%), 23F (6.4%), 6E(6B) (5.6%), 19F (5.4%) and 06A (5.3%) were the most prevalent, each accounting for over 5% of the total cohort (Fig. 1).

Within the carriage group, serotypes with frequencies greater than 5% included 19F (7.7%), 16F (6.8%), 6E(6B) (6.7%) and 23F (5.3%) (Fig. S1, available in the online Supplementary Material). The serotype distribution among carriers in Blantyre and Karonga was similar (Fig. S2). Notably, 3% of the carriage isolates (*n*=25) were identified as unencapsulated, either as Swiss_NT or untypable (Figs 1 and S1). These isolates lack the polysaccharide capsule typically used for standard serotyping, likely representing non-encapsulated strains that persist asymptomatically in the nasopharynx of healthy individuals. Swiss_NT isolates are assigned to a specific genetic lineage associated with non-encapsulated strains, while untypable isolates lack the genetic markers needed for serotype assignment and do not cluster within known serogroups. These non-encapsulated isolates were absent in blood and CSF, where only encapsulated strains can survive. The serotyping tool SeroAB assigns isolates as Swiss_NT or untypable when specific capsular gene sequences are missing or when gene variants not represented in its reference database prevent classification into known serotype groups.

Among the blood isolates, serotype 5 (20.9%) was the most dominant, followed by serotypes 1 (16.5%), 23F (8.7%) and 6A (6.1%) (Fig. S3). Among the CSF isolates, serotypes 1 (21.1%), 5 (7.7%), 12F (6.9%), 23F (6.9%) and 06A (5.4%) predominated (Fig. S4). When combining blood and CSF isolates, the most frequently observed serotypes were 1 (18.5%), 5 (15.2%), 23F (7.9%) and 06A (5.8%) (Fig. S5).

Of note, the majority of invasive samples were collected in Blantyre (94%) (Table 1). Invasive samples from Lilongwe were limited to CSF samples and exhibited a similar serotype distribution to those from Blantyre, with serotypes 1 and 12F being predominant (Fig. S6). The absence of serotype 5 in Lilongwe may be attributed to the fact that serotype 5 is more prevalent in blood, and the small number of isolates from Lilongwe (*n*=22), which were exclusively from CSF, may not have captured this serotype. Overall, the limited number of isolates from Lilongwe is insufficient to accurately represent the serotype distribution in this region.

As detailed in Table S1, serotypes 1 (*P*=9.93×10⁻³⁴), 5 (*P*=6.22×10⁻¹⁹) and 12F (*P*=2.28×10⁻⁵) were significantly overrepresented among disease cases compared to their prevalence in carriage. Given that colonization is a prerequisite for infection, these serotypes may exhibit a short nasopharyngeal colonization period before transitioning to sterile internal sites, leading to invasive disease. Serotypes 1 and 5, the most prevalent overall, are, therefore, considered the most common and highly invasive serotypes in this Malawian cohort. Based on these findings, we classify serotypes 1, 5 and 12F as ‘highly invasive’ serotypes, reflecting their significantly increased propensity to cause invasive pneumococcal disease. In contrast, serotypes 16F (*P*=8.07×10⁻⁸), 19F (*P*=1.67×10⁻⁴), 21 (*P*=8.99×10⁻⁴), 11A (*P*=1.50×10⁻^3^), Swiss_NT (*P*=1.99×10⁻³), 15C (*P*=5.27×10⁻³) and 28F (*P*=5.54×10⁻³) were significantly more prevalent among carriers than among invasive disease cases, suggesting a longer colonization rate. Consequently, these serotypes are classified as ‘highly colonized’ serotypes in this study. Serotypes 23F and 06A were found abundant in both carriage and patient groups.

**Fig. 2.**
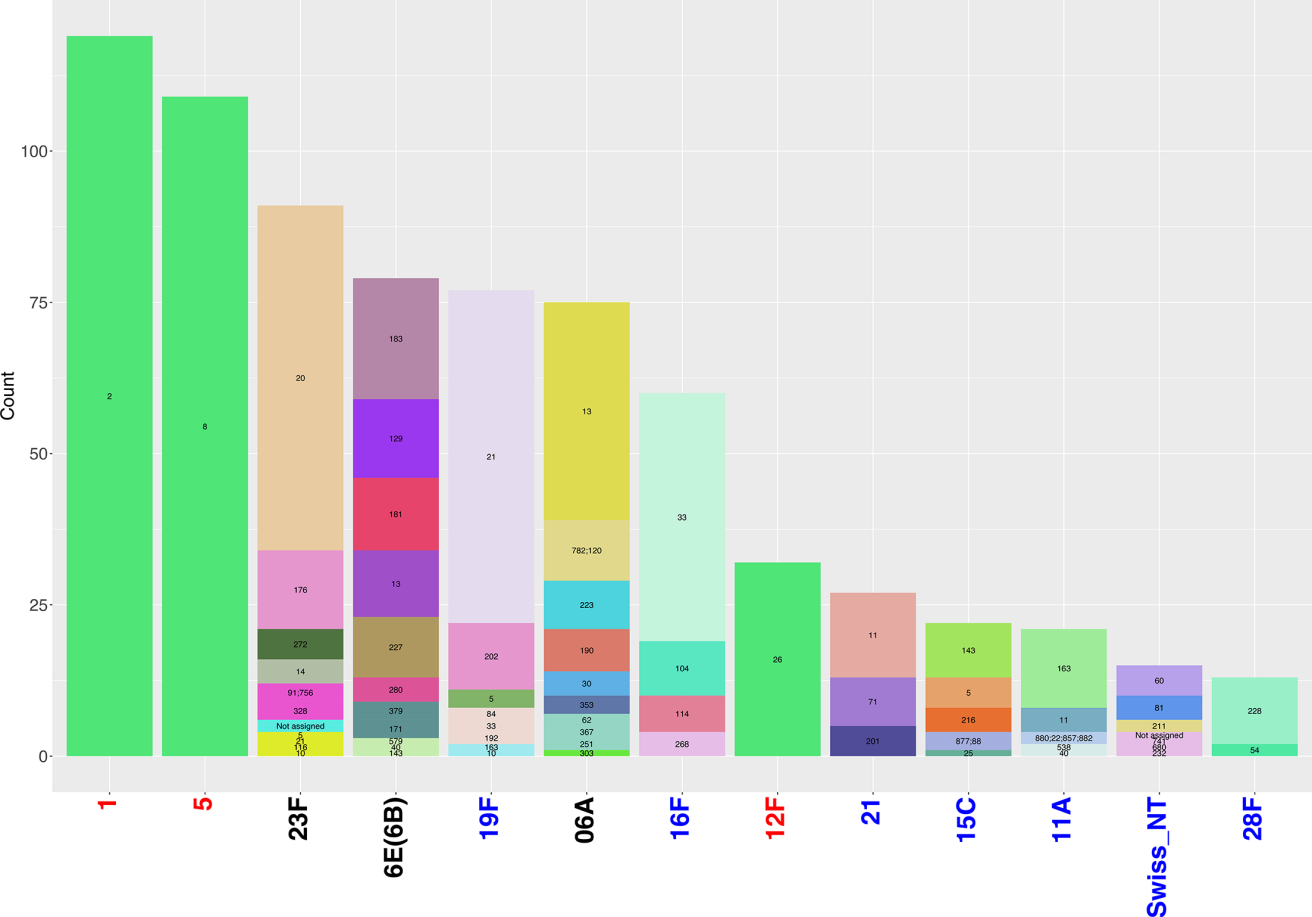
Lineage distribution within significant and abundant serotypes. Distribution of lineages within serotypes that are either significant (*P*-value<0.01) or abundant (frequency >5%) in the dataset. Serotypes are arranged by their overall prevalence. Serotypes overrepresented in the patient group are highlighted in red, and those overrepresented in the carriage group are shown in blue. Abundant serotypes with an approximately equal distribution between both groups are indicated in black. Each bar represents the abundance of a serotype, subdivided according to its constituent lineages, which are labelled within the bars. Lineages refer to GPSCs.

In addition to serotype distribution, we characterized the GPSCs and STs associated with serotypes. The dataset revealed 109 distinct GPSCs and 286 STs. Among these, only two GPSCs (2 and 8) and two STs (289 and 217) were prevalent, each exceeding a 5% frequency (Fig. S7). These abundant GPSCs and STs were primarily observed in the patient group and were associated with serotypes 1 and 5, identified as highly invasive serotypes (Figs 2 and S8). The distribution of GPSCs and STs demonstrates a notable trend: highly invasive serotypes 1, 5 and 12F are associated with a narrow genetic background, evidenced by the dominance of a single GPSC (Fig. 2).

The monoclonal nature observed for highly invasive serotypes 1, 5 and 12F suggests that specific genetic profiles may confer a selective advantage, allowing these strains to cause severe invasive disease. This advantage could be linked to optimized virulence factors. In contrast, other abundant and highly colonized serotypes exhibit greater genetic diversity by the presence of multiple GPSCs and STs. This increased genetic variability points to a more heterogeneous structure, which may reflect a higher degree of recombination, adaptive evolution or alternative strategies that may enable these serotypes to persist across various environments, including the nasopharynx, lung, blood and CSF.

### Pangenome and population structure

The genome assembly yielded an average optimized assembly hash value of 96 and an average N50 of 94 150. The mean assembled genome size was estimated at 2 115 986 nucleotides, with a standard deviation of 67 517, consistent with previously reported pneumococcal genome sizes [41]. A total of 5201 genes were identified in the pangenome, including 1463 core genes (28.13% of the pangenome), 90 soft core genes (1.73% of the pangenome), 966 shell genes (18.57% of the pangenome) and 2682 cloud genes (51.57% of the pangenome). In summary, the core genome, comprising core and soft core genes, constitutes ~30% of the pangenome, while the accessory genome, encompassing shell and cloud genes, accounts for the remaining 70%.

**Fig. 3.**
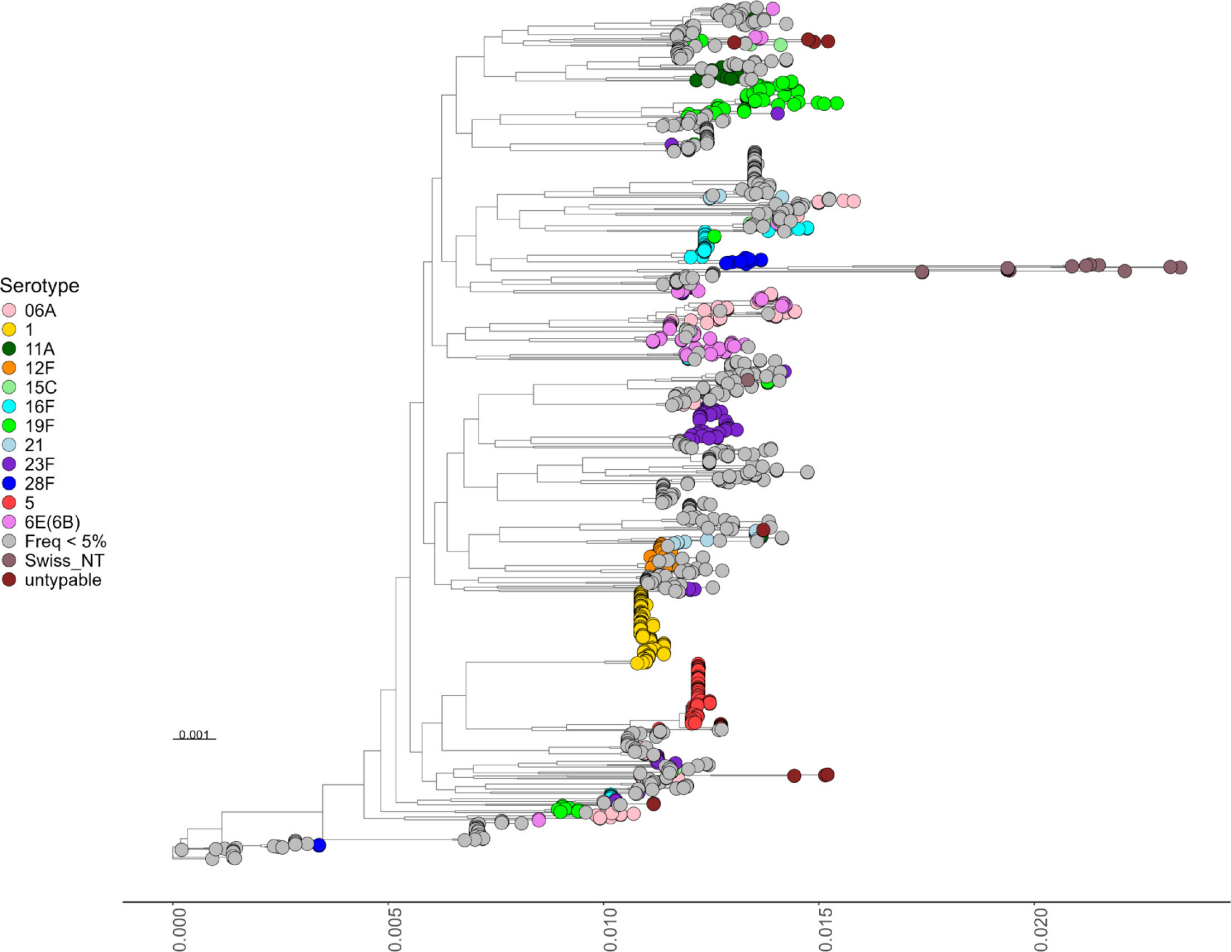
Phylogenetic tree of 1416 pneumococcal isolates from Malawi, constructed using core gene alignments and the maximum likelihood algorithm. Highly invasive serotypes 1, 5 and 12F form monophyletic clades, highlighting their monoclonal nature. Serotypes 1 and 5, the most abundant and invasive, share a common ancestry. In contrast, Swiss_NT and untypable isolates diverged significantly from the rest of the cohort, likely due to their high recombination capacity enhanced by the absence of a capsule.

The phylogenetic analysis revealed distinct clustering patterns among pneumococcal isolates (Fig. 3). Serotypes 1, 5 and 12F (highly invasive serotypes) formed monophyletic clades, indicating an evolutionary relationship within these serotypes. Specifically, serotypes 1 and 5, the most abundant and invasive serotypes, appear to have evolved from a common ancestor. This finding aligns with the observation that highly invasive serotypes are each associated with a single GPSC, further emphasizing their genetic distinctiveness and evolutionary stability as factors that may contribute to their higher invasive potential. A more conserved genetic makeup due to their monoclonal nature could possibly facilitate the conservation of key virulence factors, allowing efficient invasion and immune evasion. Stable genomes may maintain optimized variant sets for adaptation to host environments. In contrast, Swiss_NT and untypable isolates diverged from the rest of the cohort, clustering far apart. This divergence likely reflects their unique genomic architecture, including the absence or disruption of the cps locus, which eliminates the capsule. The lack of a capsule may facilitate horizontal gene transfer and genetic recombination, contributing to a more flexible accessory genome and broader genetic diversity. These isolates may also harbour paralogous genes that enhance their adaptability or contribute to virulence, providing a broader functional gene pool.

**Fig. 4.**
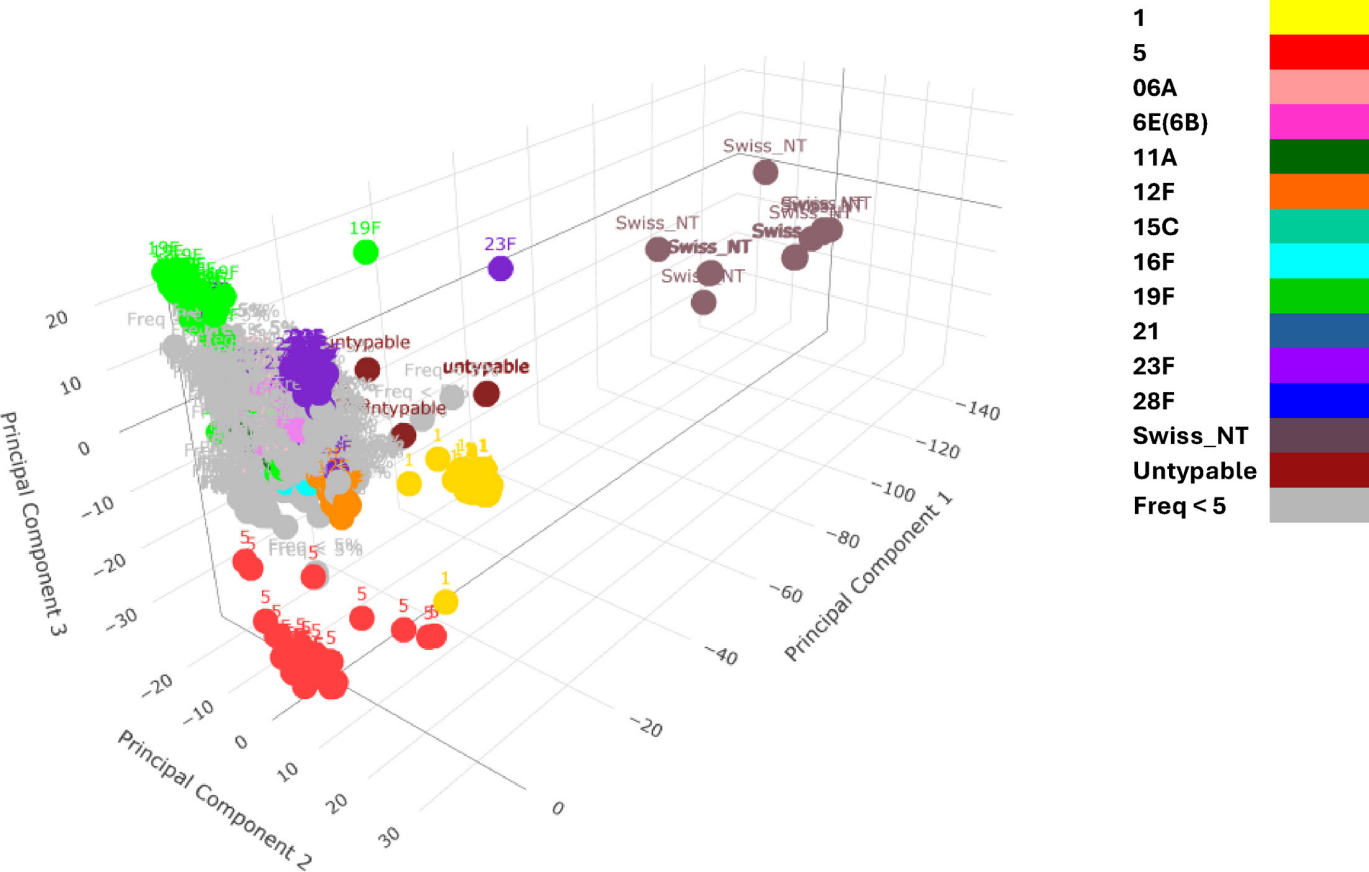
The PCA of the accessory genes’ presence–absence matrix for 1416 *Streptococcus pneumoniae* isolates from Malawi. Each point represents an isolate, coloured by serotype. The PCA reveals a clear separation of isolates based on accessory gene profiles, with highly invasive serotypes (1, 5 and 12F) and unencapsulated isolates forming distinct clusters, reflecting their diverged accessory gene content.

The PCA of accessory gene distribution demonstrated a clear separation of isolates based on accessory gene presence–absence profile (Fig. 4). Highly invasive serotypes 1, 5 and to some extent, 12F formed distinct groups and clustered separately, suggesting their accessory gene profile is distinct. An important observation is the lack of the same level of genetic distinction in serotype 12F, potentially due to its smaller sample size (*n*=32) compared to serotypes 1 (*n*=119) and 5 (*n*=109). In our dataset, serotype 12F was detected only in the post-PCV13 era, which may suggest that serotype 12F emerged as an invasive strain after the vaccination rollout. A more recent dataset could help confirm this hypothesis. Swiss_NT and moderately untypable isolates also clustered separately, showing a high degree of variability in accessory gene content. This genetic diversity is consistent with their enhanced ability to undergo genetic exchange, likely driven by the absence of a capsule, which removes a barrier to DNA uptake and recombination. Other serotypes showed lower levels of conservation compared to highly invasive serotypes, forming less distinct clusters.

Given that the majority of blood and CSF isolates were collected from Blantyre, while most nasopharyngeal samples originated from Karonga (Table 1), it is important to consider the potential impact of sampling biases. The near-complete separation of serotypes 1 and 5 from other serotypes may reflect a batch effect imposed by geographical location or their very low prevalence in the carriage group (serotype 1 : 7/119, serotype 5 : 17/109, Fig. 1), rather than genuine genetic differences. To address this potential bias, ten isolates from each highly invasive serotype and the invasive PCV13 vaccine types were randomly selected from nasopharyngeal, blood and CSF samples. A PCA of gene distribution was conducted on this downsampled dataset, which confirmed the distinct clustering patterns for serotypes 1, 5 and 12F, with each positioned apart from other known invasive serotypes (Fig. S9). This could indicate that serotypes 1, 5 and 12F are genetically distinct from other known invasive serotypes covered by PCV13, potentially contributing to the unique or more specialized pathogenic behaviours of 1, 5 and 12F, such as shorter nasopharyngeal colonization and rapid progression to blood and CSF – behaviours that, in our analysis, were not observed in other invasive vaccine types.

Another aspect to consider is the differentiation of serotypes. Serotypes are theoretically defined by their unique capsule gene compositions. However, the clustering distinctions observed for highly invasive serotypes might not be solely attributable to their distinct serotype identities because other abundant serotypes in the patient group, such as 06A and 23F, do not exhibit the same degree of genetic divergence as the highly invasive serotypes. The high abundance of 06A and 23F in the nasopharynx suggests that they may persist in the upper respiratory tract for longer periods compared to the highly invasive serotypes.

The pangenome and population structure analysis suggest that genetic divergence in highly invasive serotypes distinguishes them from other strains. This raises an important question: does this genetic distinction underlie their limited colonization capacity and accelerated infection dynamics? A functional gene presence–absence analysis could offer insights to address this question.

### Gene presence–absence analysis

As mentioned, a potential issue in the gene presence–absence association analysis between carriage and disease groups is the possible batch effect introduced by geographical locations. The samples exhibit a geographical discrepancy, with 80% of carriage samples from Karonga and 97% of disease samples from Blantyre (Table 1). This imbalance may introduce a batch effect, potentially leading to the identification of differences attributable to geographical locations rather than to the differences between non-invasive and invasive pneumococcal genomes. To further assess the potential geographical batch effect, carriage isolates from Karonga were compared with those from Blantyre. The gene presence–absence statistical test did not identify any significant differences in gene content between the Blantyre and Karonga carriage groups, indicating a similar gene profile within the carriage samples from both locations. Moreover, as previously observed, the serotype distributions in the carriage groups from Karonga and Blantyre displayed similarities (Fig. S2). Given the relatively short distance between Karonga and Blantyre and the demographic similarities between the populations, it is reasonable to expect that there would be minimal differences in the pneumococcal genomes from these two locations.

Another factor to consider is the potential presence of invasive serotypes within the carriage group, as it was not possible to determine which nasopharyngeal isolates subsequently progressed to disease after sample collection. This overlap may introduce bias in detecting invasive-associated genes through the gene presence–absence comparison between the entire carriage group and disease cases. Furthermore, the significant abundance and genetic distinction of highly invasive serotypes in the patient group skew the results of the overall carriage vs. disease analysis. To address these considerations, a gene presence–absence statistical analysis was conducted, grouping isolates based on the population structure (Figs3  4). Highly invasive serotypes, including 1, 5 and 12F, along with Swiss_NT isolates, were each compared separately to the group of highly colonized serotypes (11A, 15C, 16F, 19F, 21 and 28F). This analysis aimed to identify specific genes and pathways within the accessory genome that may have contributed to the genetic distinction between high-invasive and highly colonized serotypes, as well as to explore the high gene variability that distinguished Swiss_NT isolates from other highly colonized nasopharyngeal isolates.

The list of significant genes for each group can be found in Table S2 through Table S9.

### Gene absence profile

The functional analysis of the significant gene absence profile in highly invasive serotypes identified two enriched pathways: (i) oxidative phosphorylation and (ii) bacterial secretion system (Table 2). Functional enrichment analysis enabled the identification of significant neighbouring genes within the same chromosomal region, organized into operons. The discovery of multiple significant neighbouring genes enhances the reliability of these findings by reducing the likelihood of false positives. This is because the chance of erroneously identifying multiple neighbouring genes as significant is much lower than that of detecting a single gene with such significance.

**Table 2.** Cluster of significant neighbouring genes encoding complex components in *S. pneumoniae*

Component	Function	Presence–absence	KEGG pathway
**V-type ATP synthase**	Essential for ATP hydrolysis and proton transport, these genes form an operon to ensure coordinated expression. Their absence in certain serotypes may suggest a reduced requirement for environmental acidification	Absent from all highly invasive serotypes	Oxidative phosphorylationhttps://www.kegg.jp/entry/spn00190
**Accessory SecA2/Y2 system**	Critical for protein secretion across the cytoplasmic membrane, genes are organized in an operon. The absence indicates alternative mechanisms for protein secretion	Absent from serotypes 1, 12F and Swiss_NT. Present in serotype 5	Bacterial secretion systemhttps://www.kegg.jp/entry/spn03070

**Fig. 5.**
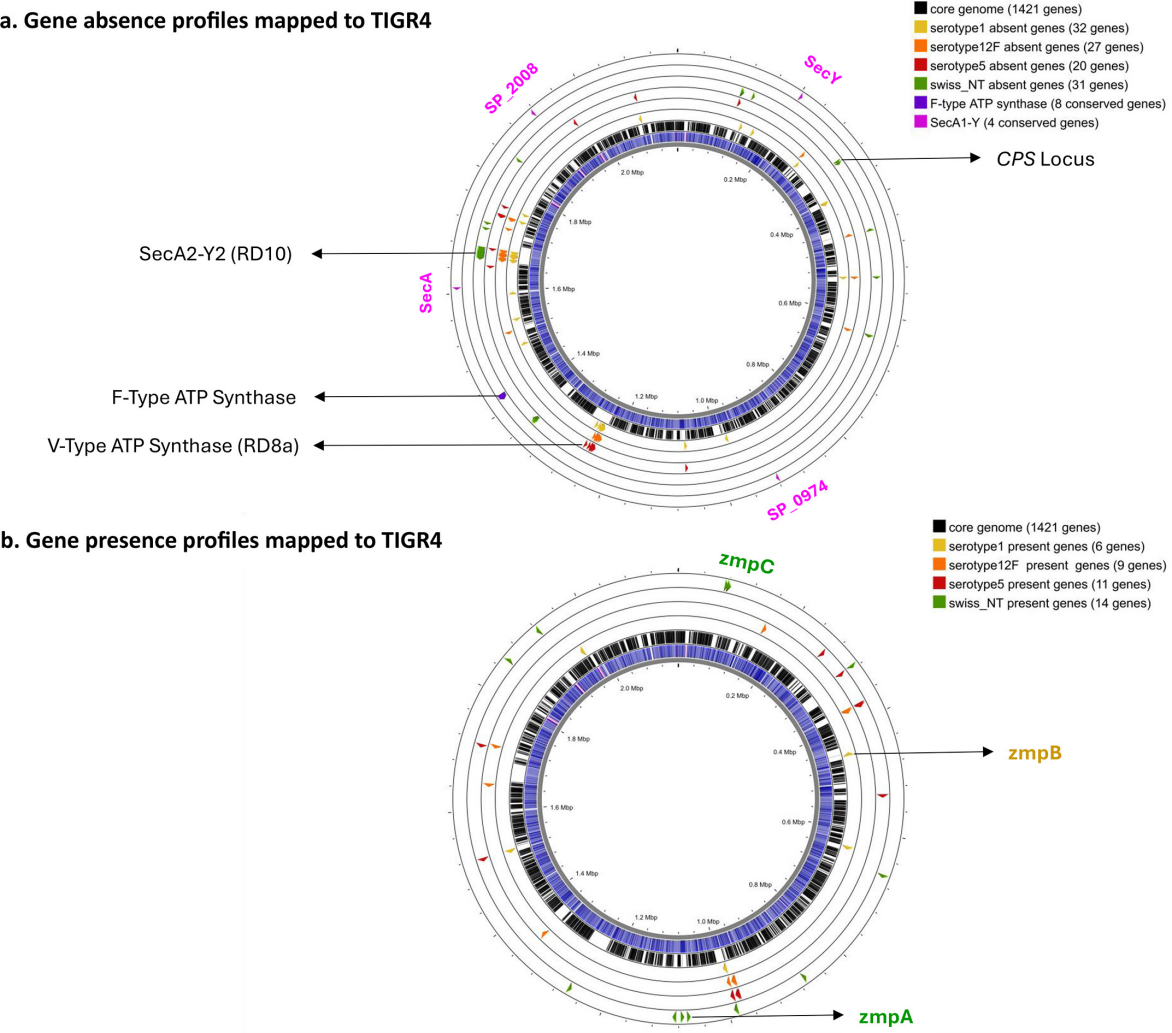
Gene presence–absence analysis. (**a**) Gene absence profiles of serotypes 1, 5, 12F and Swiss_NT mapped to the pneumococcus TIGR4 genome. The innermost blue ring represents the TIGR4 genome, followed by the location of core genes (black) in the next outer rings, where regions of diversity (RDs) appear as white blocks. The subsequent concentric rings depict genes significantly (*P*-value<0.01) absent in serotypes 1 (yellow), 12F (orange), 5 (red) and Swiss_NT (green) compared to the highly colonized serotypes (11A, 15C, 16F, 19F, 21 and 28F). Conserved core genes, including F-type ATP synthase (purple) and SecA1-Y (pink), are separately highlighted in the outermost rings. (**b**) Gene presence profiles of serotypes 1,12F, 5 and Swiss_NT, highlighted similar to panel (**a**).

Oxidative phosphorylation is linked to the function of V-type ATP synthase, a membrane-embedded ion pump that acidifies intracellular compartments by transporting protons across the bacterial membrane. This pump consists of various subunits encoded by neighbouring genes, including ntpD (SP_1315), ntpB (SP_1316), ntpA (SP_1317), ntpG (SP_1318), ntpC (SP_1319), ntpE (SP_1320) and ntpK (SP_1321). These genes are located in a region of diversity (RD) in the pneumococcus genome known as RD8a1 and were found to be absent from all highly invasive serotypes (1, 5 and 12F). In contrast, the F-type ATP synthase, homologous to the V-type ATP synthase, was identified as a core component conserved across all isolates. The core genes atpC (SP_1507), atpD (SP_1508), atpG (SP_1509), atpA (SP_1510), atpH (SP_1511), atpF (SP_1512), atpB (SP_1513) and atpE (SP_1514) encode the F-type ATP synthase (Fig. 5a). The differential distribution of V-type and F-type ATP synthases may reflect their distinct molecular functions (see the ‘Discussion’ section).

The bacterial secretion system is associated with the SecA2-SecY2 secretory channel, encoded by neighbouring genes SP_1758, secA2 (SP_1759), secY2 (SP_1763), SP_1764, SP_1765, SP_1766, SP_1767, SP_1770 and SP_1771 from RD10. These genes were significantly absent in serotypes 1 and 12F but retained in serotype 5. In *S. pneumoniae*, the SecA2-SecY2 system is involved in exporting serine-rich repeat proteins, encoded upstream (SP_1772). By contrast, the SecA1-SecY system, a homolog of SecA2-SecY2, was identified as a core component conserved across all isolates and encoded by the core genes (Fig. 5a). The absence of the SecA2-SecY2 system is unlikely to be associated with the invasive behaviour of serotypes 1 and 12F or their potential short nasopharyngeal colonization period, as we also observed its absence in unencapsulated and non-invasive Swiss_NT isolates. Instead, RD10 likely represents a recombinogenic locus in the pneumococcus genome.

As expected, the gene absence profile of Swiss_NT isolates revealed the absence of genes from the cps locus (Fig. 5a), which is responsible for capsular polysaccharide synthesis. An important finding regarding Swiss_NT and untypable isolates was that they harbour different versions of core genes in their genomes, which were classified as separate entities rather than core components by the pangenome builder Panaroo. These genes exhibited similar functions to the core genes but contained structural mutations that led to their categorization as distinct genes. This phenomenon may result from the high level of genetic recombination in unencapsulated pneumococcal strains, allowing them to maintain a repertoire of variants essential for pneumococcal fitness.

### Gene presence profile

The functional analysis of gene presence profiles in highly invasive serotypes did not reveal any enriched pathways. However, the distribution of zinc metalloproteases (zmpB, zmpA and zmpC) among isolates suggests potential associations with invasive potential (Fig. 5b). The zmpB gene was consistently present in all serotype 1 isolates but absent in isolates that persistently colonize the nasopharynx for extended periods (Table S2), raising the possibility that zmpB may contribute to promoting invasiveness rather than supporting long-term colonization. In contrast, zmpA and zmpC were fully conserved in unencapsulated Swiss_NT isolates (restricted to the nasopharynx of healthy carriers) but absent in encapsulated, highly colonized serotypes. This raises the hypothesis that zmpA and zmpC might contribute to a recombinogenic lifestyle, as their presence in Swiss_NT isolates coincided with significant enrichment of recombinase and transposase genes, potentially indicating a role in facilitating genetic recombination in these isolates (Table S8). These observations suggest the possibility of functional divergence among zinc metalloproteases, with zmpB potentially linked to invasive disease and zmpA and zmpC associated with non-invasive phenotypes. Further studies are required to validate these hypotheses and to explore the potential of targeting zmpB as part of strategies to mitigate invasive pneumococcal diseases, particularly those caused by serotype 1.

## Discussion

The *S. pneumoniae* genome is highly diverse, driven by the organism’s remarkable recombinogenic capacity. This genomic plasticity allows for the acquisition and utilization of various gene combinations, enabling *S. pneumoniae* to adapt to different habitats and respond effectively to environmental stresses. In this study, we observed 30% of the genes classified as core. These core genes, conserved across all isolates from 1997 to 2015, represent essential genetic elements critical for basic cellular functions and survival. Among the identified core components, two surface channels stood out: F-type ATP synthase and SecA1-SecY; both fully conserved across all isolates in the dataset and are located in the bacterial plasma membrane. F-type ATP synthase is essential for the organism’s survival as it generates ATP, the primary energy currency of cells. SecA1-SecY is responsible for exporting a wide range of protein types which is critical for bacteria. Their conservation across isolates from diverse GPSCs and serotypes over nearly two decades suggests their vital role in the organism’s survival. Importantly, the surface localization of these proteins highlights their potential as vaccine and therapeutic targets. Focusing on core genes for vaccine development offers a significant advantage by including unencapsulated isolates. Future research should focus on elucidating the role of the core genome in immune evasion and exploring their potential as targets for novel vaccines and drugs.

In contrast, the accessory genome displays a more sporadic and variable pattern, reflecting the genetic diversity among different strains. The presence of these accessory genes highlights the adaptability of pneumococcal strains, as they can acquire or lose genes in response to environmental pressures, such as variations in host immune responses or exposure to antibiotics.

Highly invasive serotypes 1, 5 and 12F, which show a significant prevalence among patients, were rarely found in the carrier group. This observation suggests their increased invasiveness and implies a potentially short duration of nasopharyngeal colonization. These serotypes are known to be associated with severe IPDs [42]. Serotype 1 exhibits genetic diversity across geographical regions [43] and is the leading cause of pneumococcal meningitis in Africa [44,45]. In contrast, highly colonized serotypes 11A, 15C, 16F, 19F, 21 and 28F along with unencapsulated isolates were significantly more frequent among carriers. Other abundant serotypes, such as 06A, 6E(6B), and 23F, were common among both carriers and patients, which may require a longer period of nasopharyngeal colonization before progressing to invasive infections compared to the highly invasive serotypes.

Knowing that pneumococcal virulence strongly depends on the serotype of isolates, we sought to address why several serotypes were shared across both nasopharynx and sterile sites. Here, we discuss two possible scenarios that could justify the ubiquitous and abundant presence of some serotypes among both carriages and patients. The first scenario involves the colonization of *S. pneumoniae*, which is a crucial precursor to invasion. Some samples from the carriage group may represent invasive serotypes collected during their colonization phase. The second scenario considers the mutation or differential gene expression patterns of cps genes among ubiquitous serotypes such as 06A, 6E(6B) and 23F. While cps genes were computationally identified in these serotypes from both nasopharyngeal and sterile sites, the expression of these genes may vary between these environments. Previous studies have documented a cycle of encapsulation and de-encapsulation in pneumococcal strains. Isolates can benefit from mutations in the cps locus or alter gene expression to either halt or resume capsule production [46]. Therefore, the presence of the cps locus in the genomes of isolates does not necessarily indicate that all cells are encapsulated.

In this study, unencapsulated isolates refer to isolates that lack the cps locus, which is critical for classification into specific serotypes. These isolates (Swiss_NT and untypable) were exclusively detected in the nasopharynx. This lack of a capsule impairs their ability to evade detection in sterile sites like blood and CSF, leading to their predominant presence in the nasopharyngeal region of carriers. Moreover, the absence of a capsule at the epithelial surface allows the bacterium to expose its surface proteins, promoting adherence to host epithelial cells. It is estimated that 15% of isolates in the upper respiratory tract are unencapsulated and adhere more efficiently than their encapsulated counterparts [47]. Moreover, the lack of a capsule facilitates the acquisition of virulence and resistance genes from other strains during colonization.

The study highlights the significant genetic distinction of highly invasive serotypes by identifying their monoclonal nature and the presence or absence of genes in their genomic structure compared to highly colonized serotypes. Several genes with specific functions were identified, which may shed light on the biological basis of high invasiveness. However, the findings suggest that the gene presence–absence profiles alone cannot fully explain the highly invasive properties of serotypes such as 1, 5 and 12F, although they do offer some insight into evolutionary pressures. A primary challenge in analysing the gene presence–absence within the pneumococcal genome lies in the observation that many genes significantly detected present/absent in highly invasive serotypes (1, 5 and 12F) also have homologs present/absent in other isolates or within the core genome. This can dilute the potential link between the functional significance of these genes and the invasiveness of specific serotypes. The invasiveness of these serotypes may instead be influenced by factors such as synteny, gene copy number or differential gene expression. Moreover, many of the significant genes identified are either uncharacterized or annotated as hypothetical proteins, underscoring the critical need for comprehensive gene annotation to better understand their roles.

One of the findings of this study is the absence of V-type ATP synthase in highly invasive serotypes, while F-type ATP synthase was found to be a conserved core component across all isolates. This difference in the distribution of F-type and V-type ATP synthases may reflect their different functional roles in pneumococcus. F-type ATP synthase is essential for ATP production, utilizing the proton motive force to convert ADP and inorganic phosphate into ATP. Conversely, V-type ATP synthase (V-ATPase) primarily functions as a proton pump, using ATP hydrolysis to acidify the surrounding environment [48,49]. The ubiquitous presence of F-type ATP synthase emphasizing its critical role in fundamental cellular processes, such as energy generation, positions it as a potential therapeutic target. In contrast, V-type ATP synthase may provide strain-specific advantages, such as acidifying the environment to outcompete other microorganisms or enhance colonization capabilities [50]. Highly invasive serotypes appear to exhibit reduced reliance on the ion-pumping functions of V-type ATP synthase. However, linking this absence to their rapid infectious properties and low colonization rates remains speculative and requires further investigation. Understanding the potential role of V-type ATP synthase in these serotypes could help clarify the mechanisms underlying their invasive behaviour and transient colonization.

Proteins in bacteria are exported via secretion (Sec) systems. In Gram-positive bacteria, including *S. pneumoniae*, two main types of general Sec systems are present: (i) the canonical SecA1-SecY(E/G) system, which consists of the ATPase SecA1 and the membrane-spanning complex SecY(E/G), and (ii) the SecA2-SecY2 system, which includes the ATPase SecA2 and the SecY2 channel and operates alongside a set of glycosyltransferases (Gtfs) [50]. In *S. pneumoniae*, the SecA1-SecY(E/G) system is responsible for exporting a wide range of protein types, whereas the SecA2-SecY2 system exports serine-rich proteins, which are glycosylated by Gtfs before secretion. The precise mechanism of the SecA2-SecY2 system is not fully understood. In *S. pneumoniae*, the SecA2-SecY2 system shares homology with the SecA2-SecY2 system in *Streptococcus gordonii*, which facilitates the secretion of protein B, linked to infective endocarditis [51]. Research on *S. gordonii* has indicated that the presence of SecA2-SecY2 may facilitate the export of pneumolysin and adhesion to both epithelial cells in the nasopharynx and erythrocytes in the blood [52,53]. Similarly, the SecA2-SecY2 system may also be beneficial for pneumococcal isolates in the nasopharynx (highly colonized) and serotype 5, which is the most abundant in the blood. However, further research is required to explain why this system is absent in serotypes like 1 and 12F as well as Swiss_NTs.

In conclusion, the differences in gene composition between highly invasive and highly colonized serotypes highlight the role of genetic factors in determining the pathogenic potential of pneumococcal strains. This emphasizes the importance of ongoing surveillance to monitor changes in serotype distribution and their epidemiological impact. Our research revealed significant genetic divergence in highly invasive serotypes in Malawi, which may be associated with their rapid progression to invasive disease. Further investigation, including high-throughput techniques like gene expression analysis, could provide deeper insights into the differences between carriage and invasive isolates, particularly for serotypes prevalent in both. Overall, this study provides insight into the pneumococcal population structure and serotype distribution in Malawi. The significant genes identified in highly invasive serotypes, along with highly conserved core genes, could serve as targets for future therapeutic interventions. Experimental validation is needed to confirm the computational findings from this study.

## Supplementary material

10.1099/mgen.0.001370 Uncited Supplementary Material 1.

10.1099/mgen.0.001370 Uncited Supplementary Material 2.

## Abbreviations

cps: capsular polysaccharide
CSF: cerebrospinal fluid
GPSCs: global pneumococcal sequence clusters
Gtfs: glycosyltransferases
IPDs: invasive pneumococcal diseases
MLST: multilocus sequence typing
MLW: Malawi-Liverpool Wellcome Trust Clinical Research Programme
OR: odds ratio
PCA: principal component analysis
PCV: pneumococcal conjugate vaccine
RD: region of diversity
Sec: secretion
SRST2: short read sequence typing 2
ST: sequence type

## References

[1] (2025). Pneumococcal disease. https://www.who.int/teams/health%20product-policy-and-standards/standards-and-specifications/norms-and-standards/vaccine%20standardization/pneumococcal-disease?utm_source=chatgpt.com.

[2] Kamthunzi P (2021). Impact of PCV13 vaccination in Blantyre, Malawi. Lancet Glob Health.

[3] Bogaert D, De Groot R, Hermans PWM (2004). *Streptococcus pneumoniae* colonisation: the key to pneumococcal disease. *Lancet Infect Dis*.

[4] Backhaus E, Berg S, Andersson R, Ockborn G, Malmström P (2016). Epidemiology of invasive pneumococcal infections: manifestations, incidence and case fatality rate correlated to age, gender and risk factors. BMC Infect Dis.

[5] Kadioglu A, Weiser JN, Paton JC, Andrew PW (2008). The role of *Streptococcus pneumoniae* virulence factors in host respiratory colonization and disease. *Nat Rev Microbiol*.

[6] Weiser JN, Ferreira DM, Paton JC (2018). *Streptococcus pneumoniae*: transmission, colonization and invasion. *Nat Rev Microbiol*.

[7] Orihuela CJ, Gao G, Francis KP, Yu J, Tuomanen EI (2004). Tissue-specific contributions of pneumococcal virulence factors to pathogenesis. J Infect Dis.

[8] Nelson AL, Roche AM, Gould JM, Chim K, Ratner AJ (2007). Capsule enhances pneumococcal colonization by limiting mucus-mediated clearance. Infect Immun.

[9] Geno KA, Gilbert GL, Song JY, Skovsted IC, Klugman KP (2015). Pneumococcal capsules and their types: past, present, and future. Clin Microbiol Rev.

[10] Ganaie F, Saad JS, McGee L, van Tonder AJ, Bentley SD (2020). A new pneumococcal capsule type, 10D, is the 100th serotype and has a large cps fragment from an oral *Streptococcus*. mBio.

[11] Weinberger DM, Malley R, Lipsitch M (2011). Serotype replacement in disease after pneumococcal vaccination. Lancet.

[12] Feikin DR, Kagucia EW, Loo JD, Link-Gelles R, Puhan MA (2013). Serotype-specific changes in invasive pneumococcal disease after pneumococcal conjugate vaccine introduction: a pooled analysis of multiple surveillance sites. PLoS Med.

[13] McCollum ED, Nambiar B, Deula R, Zadutsa B, Bondo A (2017). “Impact of the 13-valent pneumococcal conjugate vaccine on clinical and hypoxemic childhood pneumonia over three years in central Malawi: an observational study. PLoS One.

[14] Heinsbroek E, Tafatatha T, Phiri A, Swarthout TD, Alaerts M (2018). Pneumococcal carriage in households in Karonga District, Malawi, before and after introduction of 13-valent pneumococcal conjugate vaccination. Vaccine.

[15] Bar-Zeev N, Swarthout TD, Everett DB, Alaerts M, Msefula J (2020). Impact and effectiveness of 13-valent pneumococcal conjugate vaccine on population incidence of vaccine and non-vaccine serotype invasive pneumococcal disease in Blantyre, Malawi, 2006-2018: prospective observational time-series and case-control studies. SSRN J.

[16] Appelbaum PC (2002). Resistance among *Streptococcus pneumoniae*: implications for drug selection. Clin Infect Dis.

[17] Iranzadeh A, Mulder NJ (2019). Microbial Genomics in Sustainable Agroecosystems.

[18] Kamng’ona AW, Hinds J, Bar-Zeev N, Gould KA, Chaguza C (2015). High multiple carriage and emergence of *Streptococcus pneumoniae* vaccine serotype variants in malawian children. BMC Infect Dis.

[19] Chaguza C, Cornick JE, Andam CP, Gladstone RA, Alaerts M (2017). Population genetic structure, antibiotic resistance, capsule switching and evolution of invasive pneumococci before conjugate vaccination in Malawi. Vaccine.

[20] Andrews S (2010). FastQC: a quality control tool for high throughput sequence data. Babraham Bioinformatics.

[21] Ewels P, Magnusson M, Lundin S, Käller M (2016). MultiQC: summarize analysis results for multiple tools and samples in a single report. Bioinformatics.

[22] Gladman S, Seemann T (2008). VelvetOptimiser.

[23] Gurevich A, Saveliev V, Vyahhi N, Tesler G (2013). QUAST: quality assessment tool for genome assemblies. Bioinformatics.

[24] Seemann T (2014). Prokka: rapid prokaryotic genome annotation. Bioinformatics.

[25] Epping L, van Tonder AJ, Gladstone RA, Bentley SD, Page AJ (2018). SeroBA: rapid high-throughput serotyping of *Streptococcus pneumoniae* from whole genome sequence data. Microb Genom.

[26] Lees JA (2019). Fast and flexible bacterial genomic epidemiology with poppunk. Genome Res.

[27] Inouye M, Dashnow H, Raven L-A, Schultz MB, Pope BJ (2014). SRST2: rapid genomic surveillance for public health and hospital microbiology labs. Genome Med.

[28] Argimón S, David S, Underwood A, Abrudan M, Wheeler NE (2021). Rapid genomic characterization and global surveillance of *Klebsiella* using pathogenwatch. Clin Infect Dis.

[29] Tonkin-Hill G, MacAlasdair N, Ruis C, Weimann A, Horesh G (2020). SOFTWARE open access producing polished prokaryotic pangenomes with the Panaroo pipeline. Genome Biol.

[30] Minh BQ, Schmidt HA, Chernomor O, Schrempf D, Woodhams MD (2020). IQ-TREE 2: new models and efficient methods for phylogenetic inference in the genomic era. Mol Biol Evol.

[31] Wickham H (2011). ggplot2. WIREs Computational Stats.

[32] Yu G, Smith DK, Zhu H, Guan Y, Lam TTY (2017). Ggtree: an r package for visualization and annotation of phylogenetic trees with their covariates and other associated data. Methods Ecol Evol.

[33] Rohart F, Gautier B, Singh A, Lê Cao KA (2017). mixOmics: an R package for 'omics feature selection and multiple data integration. PLoS Comput Biol.

[34] Interactive web-based data visualization with R, plotly, and shiny. https://plotly-r.com/.

[35] Brynildsrud O, Bohlin J, Scheffer L, Eldholm V (2016). Rapid scoring of genes in microbial pan-genome-wide association studies with Scoary. Genome Biol.

[36] Camacho C, Coulouris G, Avagyan V, Ma N, Papadopoulos J (2009). BLAST+: architecture and applications. BMC Bioinf.

[37] Szklarczyk D, Kirsch R, Koutrouli M, Nastou K, Mehryary F (2023). The STRING database in 2023: protein-protein association networks and functional enrichment analyses for any sequenced genome of interest. Nucleic Acids Res.

[38] Ogata H, Goto S, Sato K, Fujibuchi W, Bono H (1999). KEGG: kyoto encyclopedia of genes and genomes. Nucleic Acids Res.

[39] Bateman A, Coin L, Durbin R, Finn RD, Hollich V (2004). The Pfam protein families database. Nucleic Acids Res.

[40] Grant JR, Enns E, Marinier E, Mandal A, Herman EK (2023). Proksee: in-depth characterization and visualization of bacterial genomes. Nucleic Acids Res.

[41] Hiller NL (2007). Comparative genomic analyses of seventeen *Streptococcus pneumoniae* strains: insights into the pneumococcal supragenome. J Bacteriol.

[42] Johnson HL, Deloria-Knoll M, Levine OS, Stoszek SK, Freimanis Hance L (2010). Systematic evaluation of serotypes causing invasive pneumococcal disease among children under five: the pneumococcal global serotype project. PLoS Med.

[43] Cornick JE, Chaguza C, Harris SR, Yalcin F, Senghore M (2015). Region-specific diversification of the highly virulent serotype 1 *Streptococcus pneumoniae*. Microb Genom.

[44] Leimkugel J, Adams Forgor A, Gagneux S, Pflüger V, Flierl C (2005). An outbreak of serotype 1 *Streptococcus pneumoniae* meningitis in northern Ghana with features that are characteristic of *Neisseria meningitidis* meningitis epidemics. J Infect Dis.

[45] Gessner BD, Mueller JE, Yaro S (2010). African meningitis belt pneumococcal disease epidemiology indicates a need for an effective serotype 1 containing vaccine, including for older children and adults. BMC Infect Dis.

[46] Langereis JD, de Jonge MI (2017). Non-encapsulated *Streptococcus pneumoniae*, vaccination as a measure to interfere with horizontal gene transfer. Virulence.

[47] Talbot UM, Paton AW, Paton JC (1996). Uptake of *Streptococcus pneumoniae* by respiratory epithelial cells. Infect Immun.

[48] Boyer PD (1997). The ATP synthase—a splendid molecular machine. Annu Rev Biochem.

[49] Cross RL, Müller V (2004). The evolution of A-, F-, and V-type ATP synthases and ATPases: reversals in function and changes in the H+/ATP coupling ratio. FEBS Lett.

[50] Feltcher ME, Braunstein M (2012). Emerging themes in SecA2-mediated protein export. Nat Rev Microbiol.

[51] Bensing BA, Gibson BW, Sullam PM (2004). The *Streptococcus gordonii* platelet binding protein GspB undergoes glycosylation independently of export. J Bacteriol.

[52] Bensing BA, Sullam PM (2010). Transport of preproteins by the accessory sec system requires a specific domain adjacent to the signal peptide. J Bacteriol.

[53] Yamaguchi M, Terao Y, Mori-Yamaguchi Y, Domon H, Sakaue Y (2013). *Streptococcus pneumoniae* invades erythrocytes and utilizes them to evade human innate immunity. PLoS One.

