# Supplementary material for: Comparative pangenomics of Streptococcus pneumoniae from Malawi: uncovering genetic variability and pathogenicity
Source: Microb Genom. 2025 Apr 15;11(4):001370. doi: 10.1099/mgen.0.001370 (PMC12282314; doi:10.1099/mgen.0.001370)
Supplement: Uncited Supplementary Material 1. [file mgen-11-01370-s001.pdf]

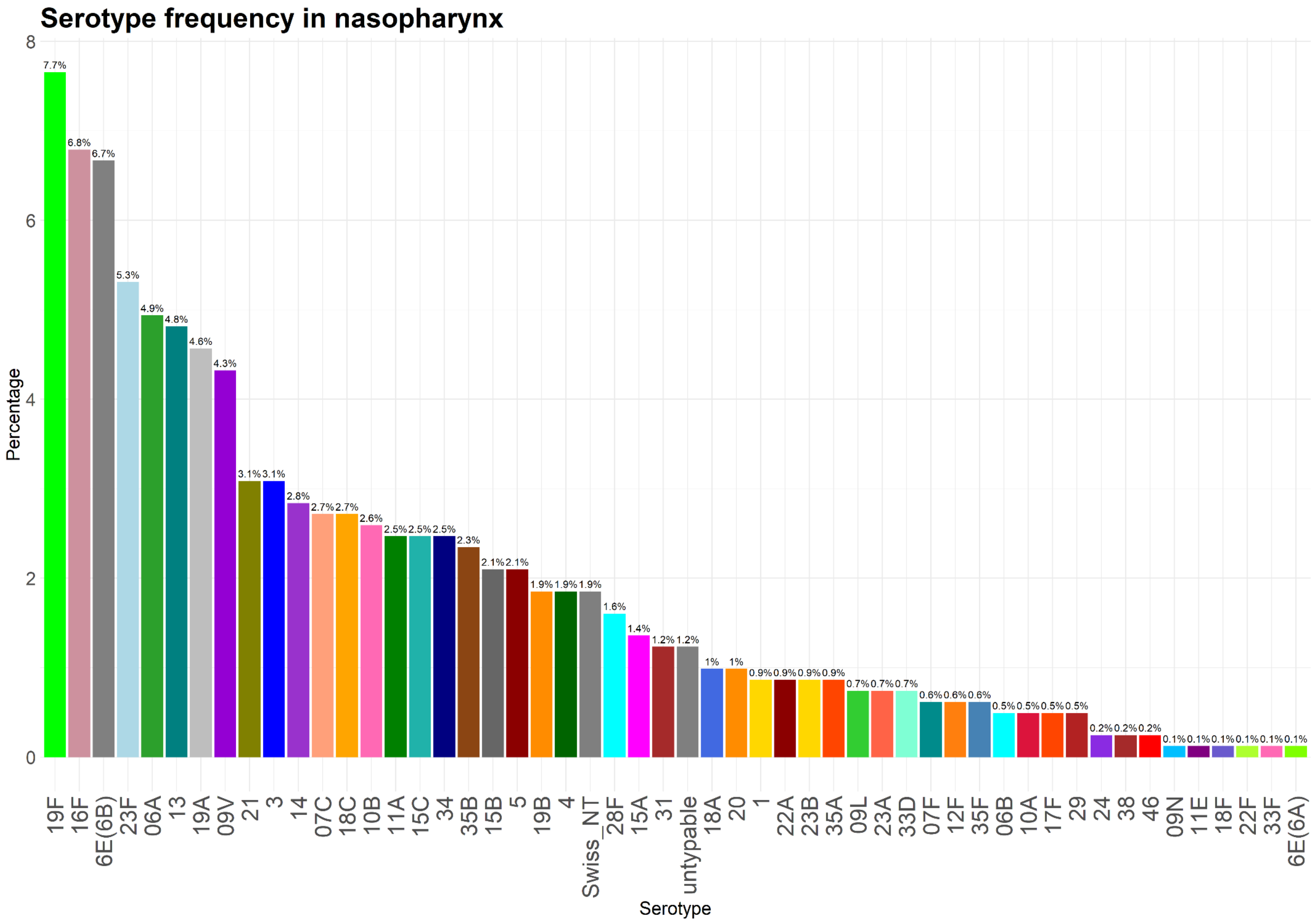

S1 Fig. Serotype distribution in the carriage group. The frequency of each serotype in the carriage group is denoted above each bar. The abundant serotypes (relative frequency > 5%) were 19F (7.7%), 16F (6.8%), 6E(6B) (6.7%), and 23F (5.3%).

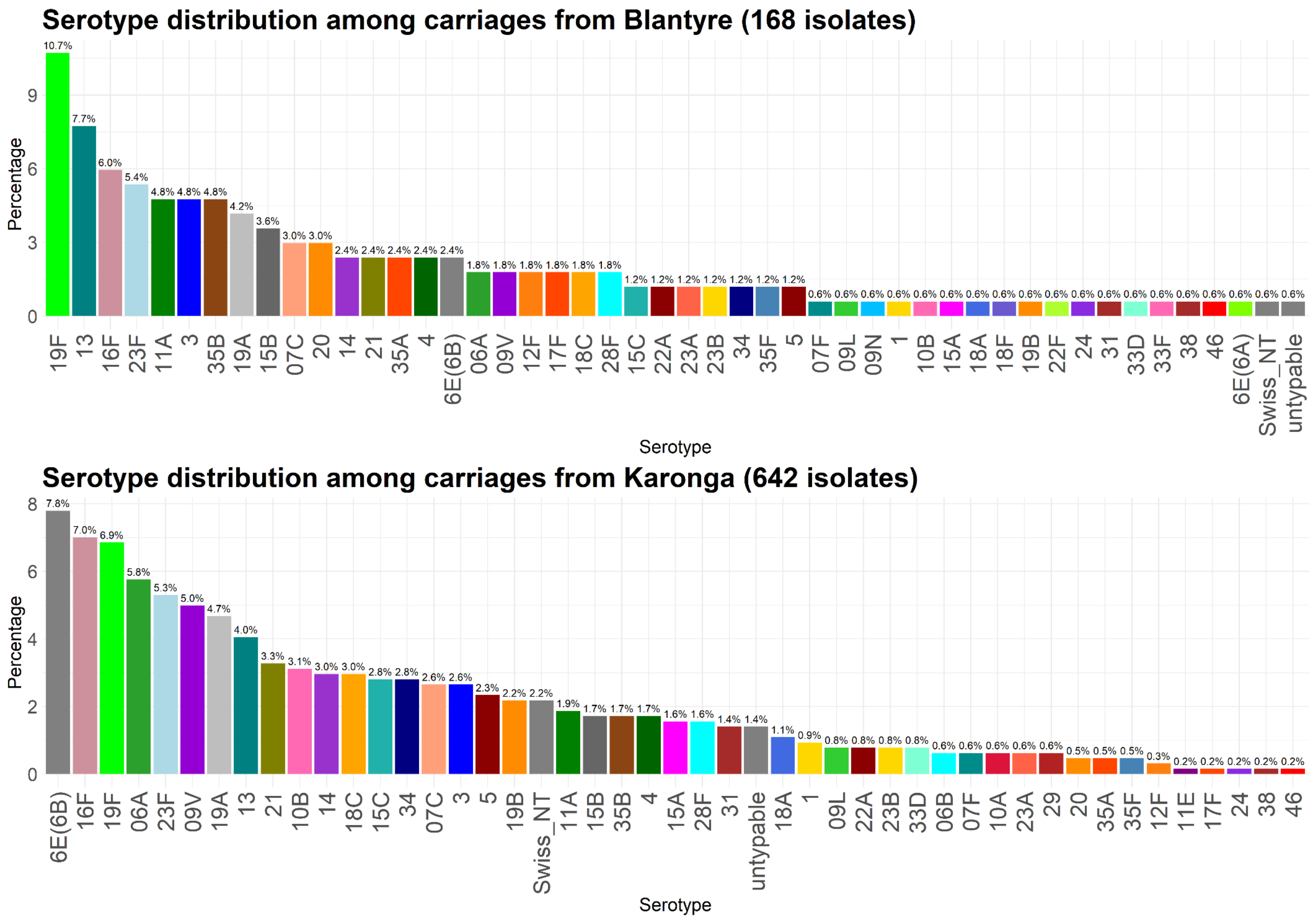

S2 Fig. Comparison of serotype distributions among carriage isolates in Karonga and Blantyre. The distribution of abundant serotypes in Karonga and Blantyre was largely similar. Sserotype 13 was slightly more prevalent in Blantyre, and serotype 6E(6B), 06A, and 09V was detected more in Karonga.

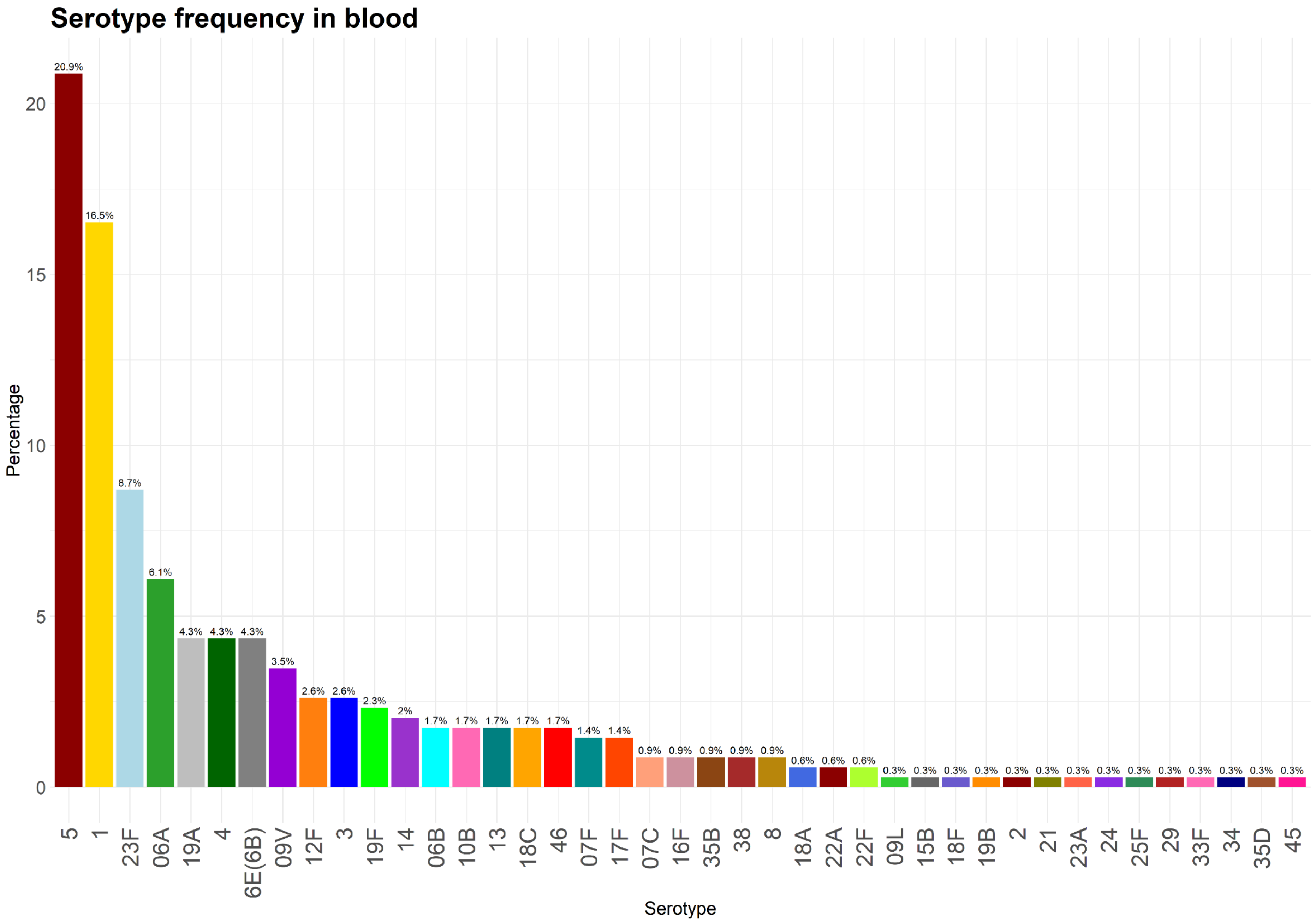

S3 Fig. Serotype distribution in blood samples. The frequency of each serotype in blood samples is indicated above each bar. The most abundant serotypes (relative frequency > 5%) were serotype 5 (20.9%), serotype 1 (16.5%), serotype 23F (8.7%), and serotype 06A (6.1%).

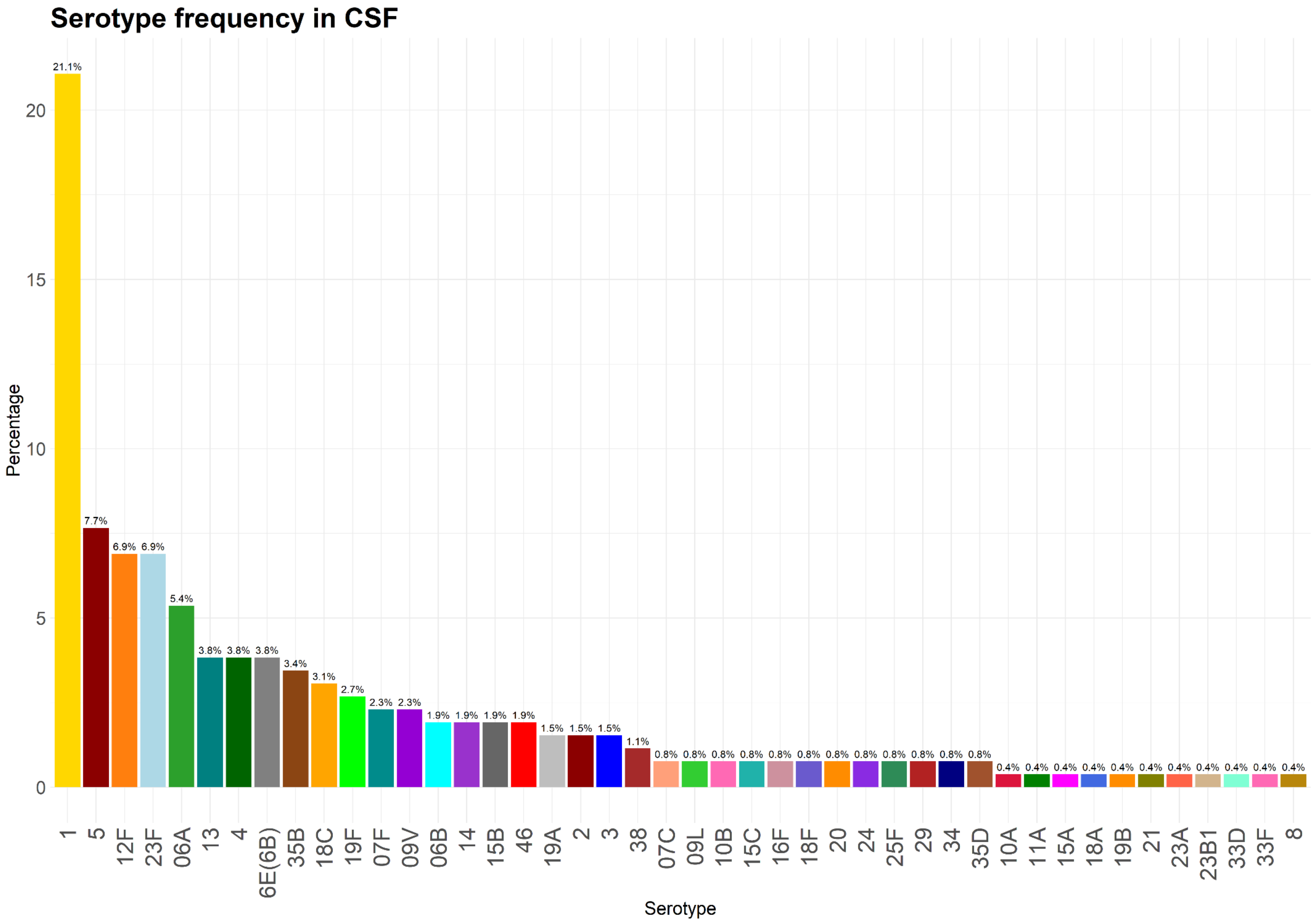

S4 Fig. Serotype distribution in cerebrospinal fluid (CSF) samples. The frequency of each serotype in CSF samples is indicated above each bar. The most abundant serotypes (relative frequency > 5%) were serotype 1 (21.1%), serotype 5 (7.7%), serotype 12F (6.9%), serotype 23F (6.9%), and serotype 06A (5.4%).

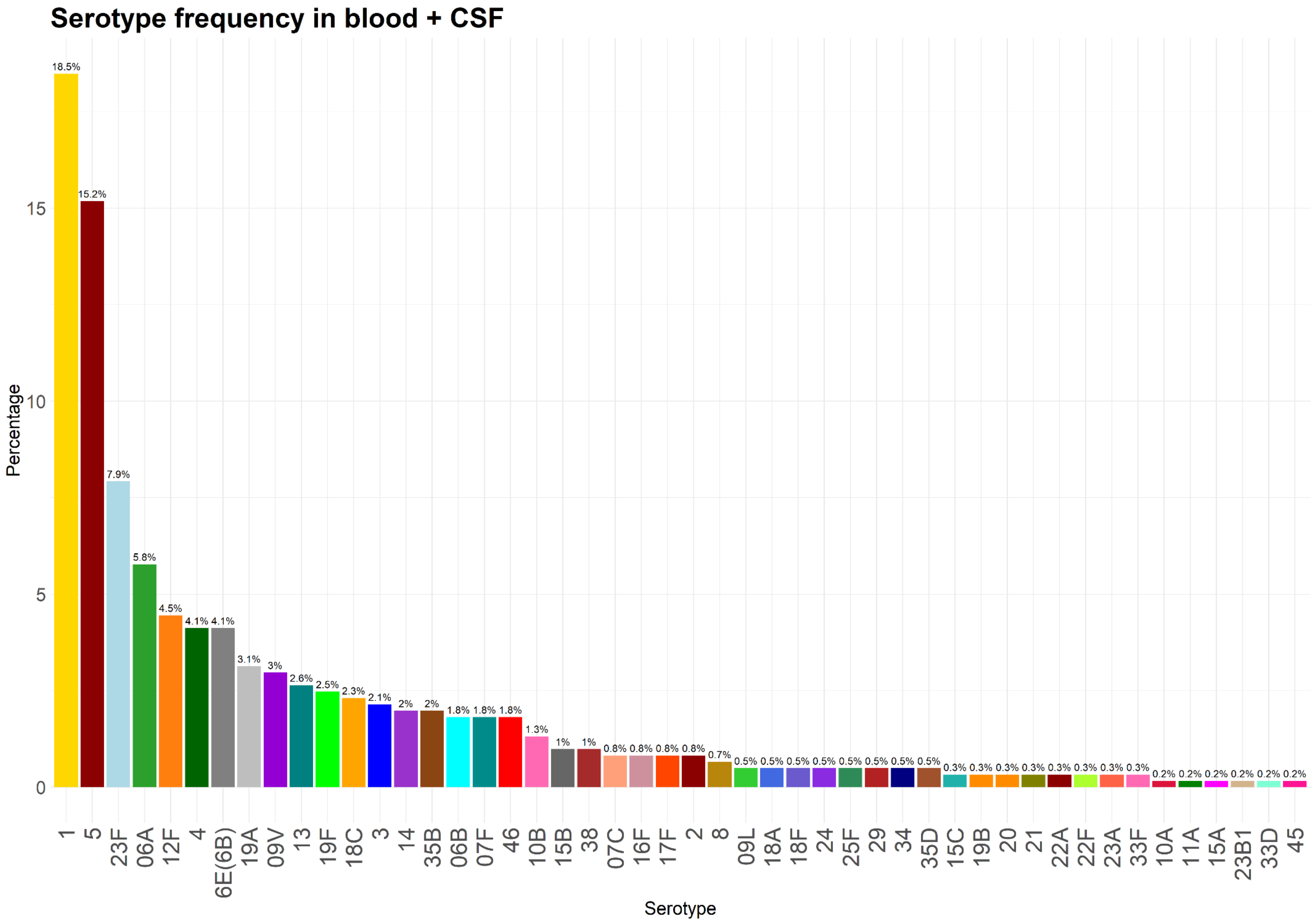

S5 Fig. Serotype distribution in cerebrospinal fluid (CSF) and blood samples. The frequency of each serotype in blood and CSF samples is indicated above each bar. The most abundant serotypes (relative frequency > 5%) were serotype 1 (18.5%), serotype 5 (15.2%), serotype 23F (7.9%), and serotype 06A (5.8%).

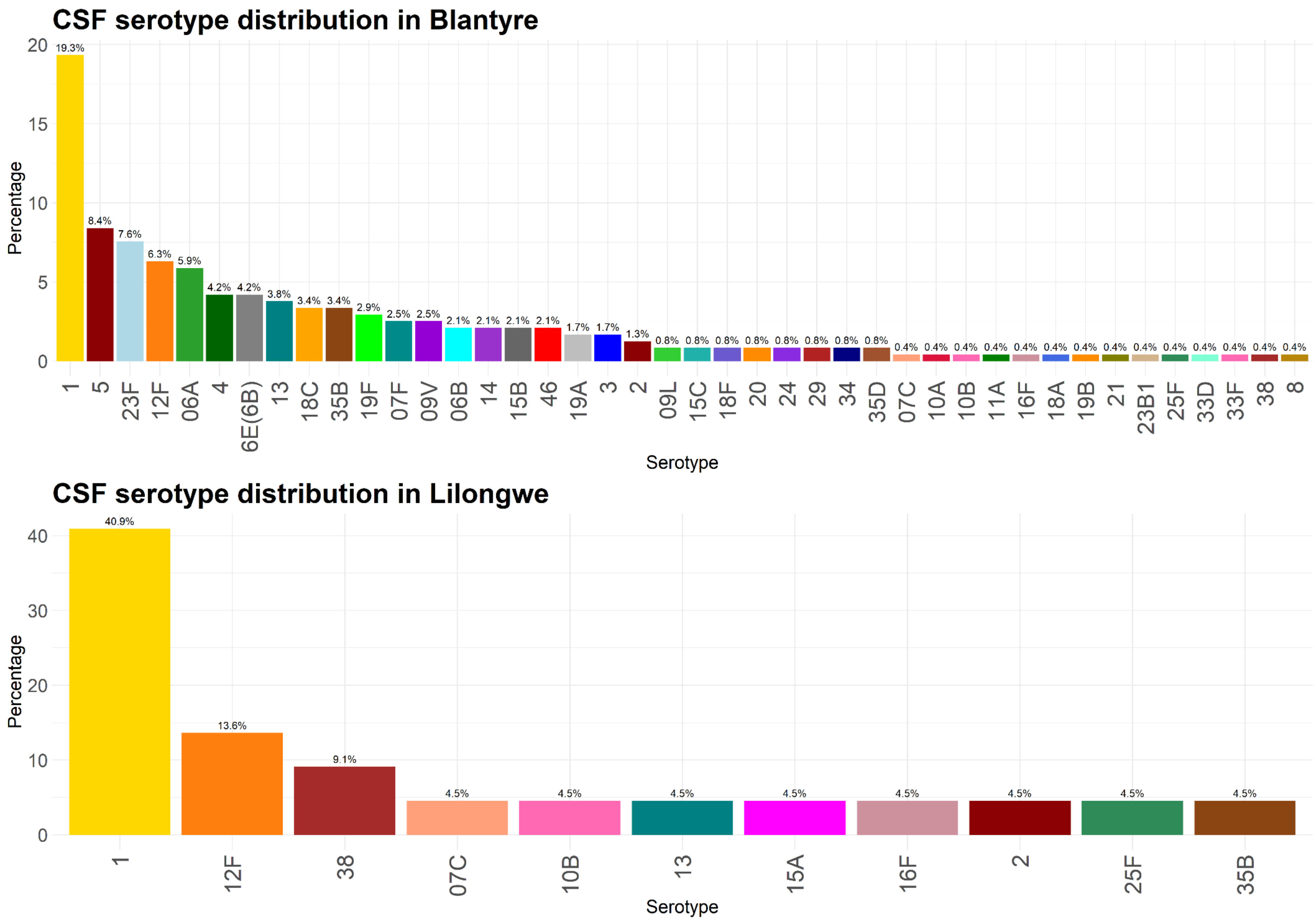

S6 Fig. Serotype distribution in cerebrospinal fluid (CSF) samples from Blantyre and Lilongwe. Only 3.5% of disease samples were collected from Lilongwe. However, serotypes 1 and 12F were abundant (relative frequency > 5%) in both regions. A larger dataset from Lilongwe may provide a more accurate reflection of the serotype distribution in this area.

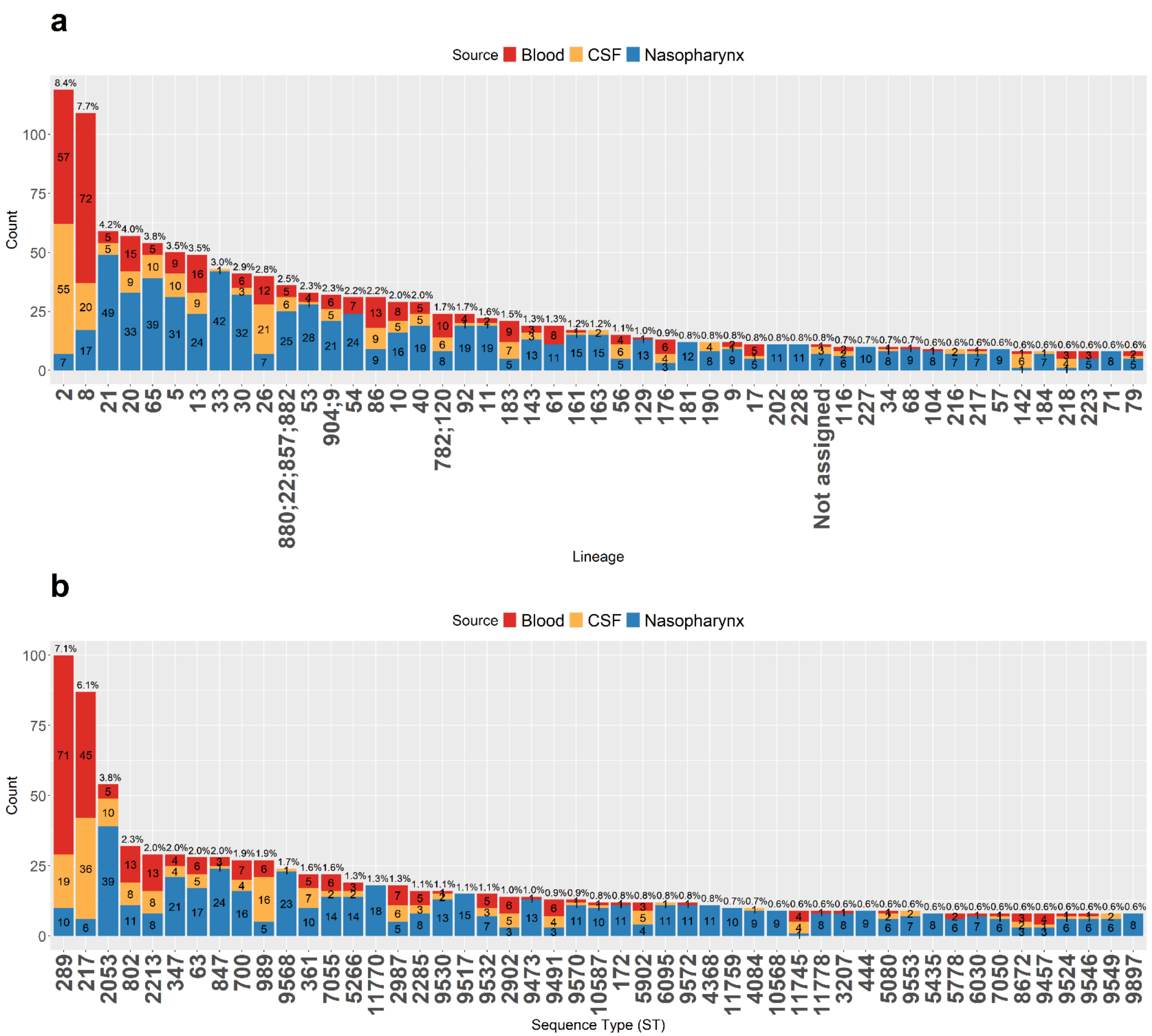

S7 Fig. Distribution of (a) lineages and (b) Sequence Types (STs) in the dataset. A total of 109 lineages and 286 STs were identified. The most prevalent lineages were 2 and 8, while the most abundant STs were 289 and 217, primarily observed in the patient group. For clarity, lineages and STs with a relative frequency below 0.5% were excluded from the figures. Lineages refer to Global Pneumococcal Sequence Clusters (GPSCs).

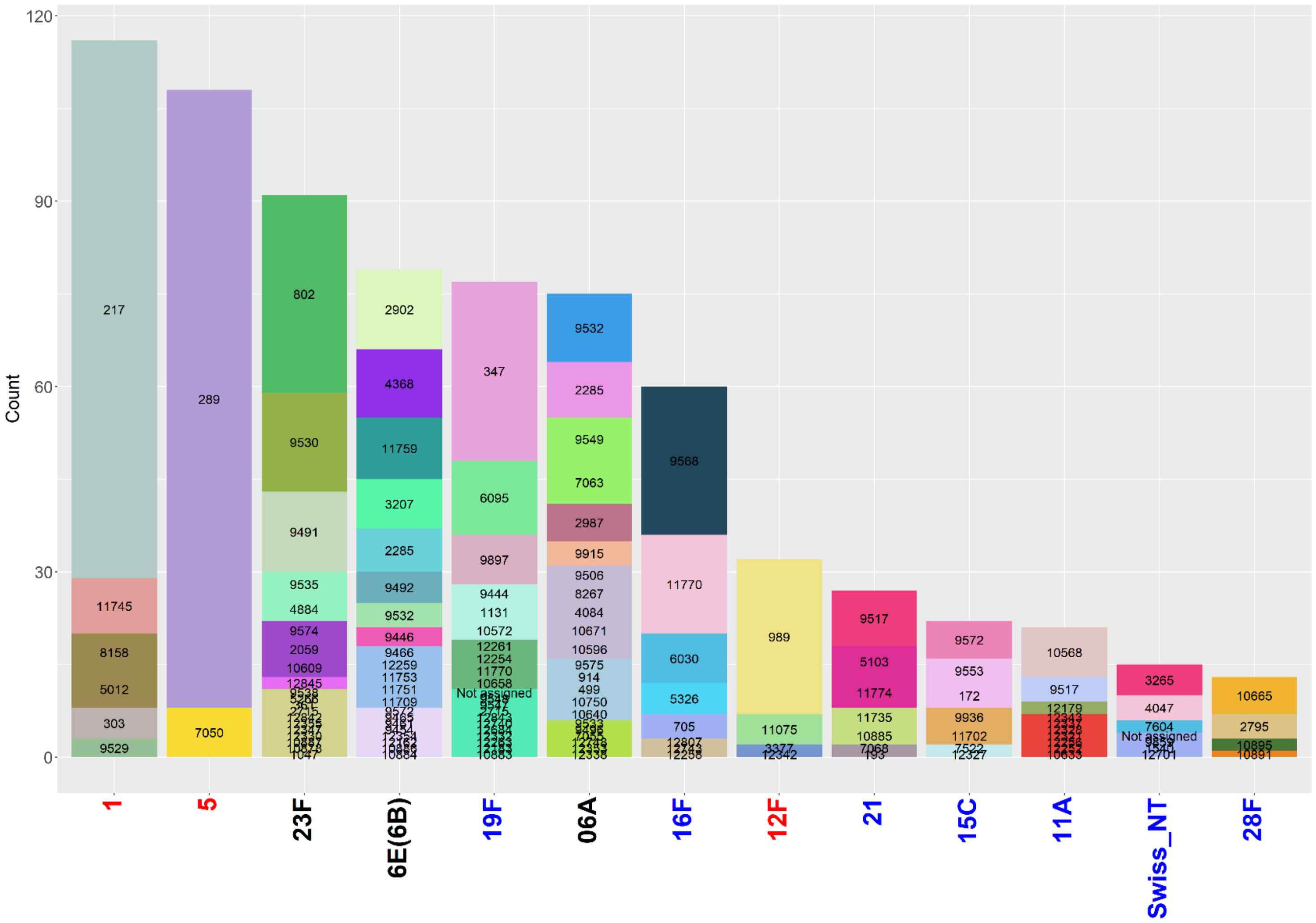

S8 Fig. Sequence Type (ST) distribution within significant and abundant serotypes. Distribution of STs within serotypes that are either significant or abundant in the dataset. Serotypes are arranged by their overall prevalence. Fisher's exact test with a Benjamini-Hochberg (BH)-adjusted p-value threshold of < 0.01 was applied to identify serotypes with significantly different distributions between the carriage and patient groups. Serotypes overrepresented in the patient group are highlighted in red and those overrepresented in the carriage group are shown in blue. Serotypes with a prevalence exceeding 5% and approximately equal distribution between both groups are indicated in black. Each bar represents the abundance of a serotype, divided according to its constituent STs, which are labelled within the bars.

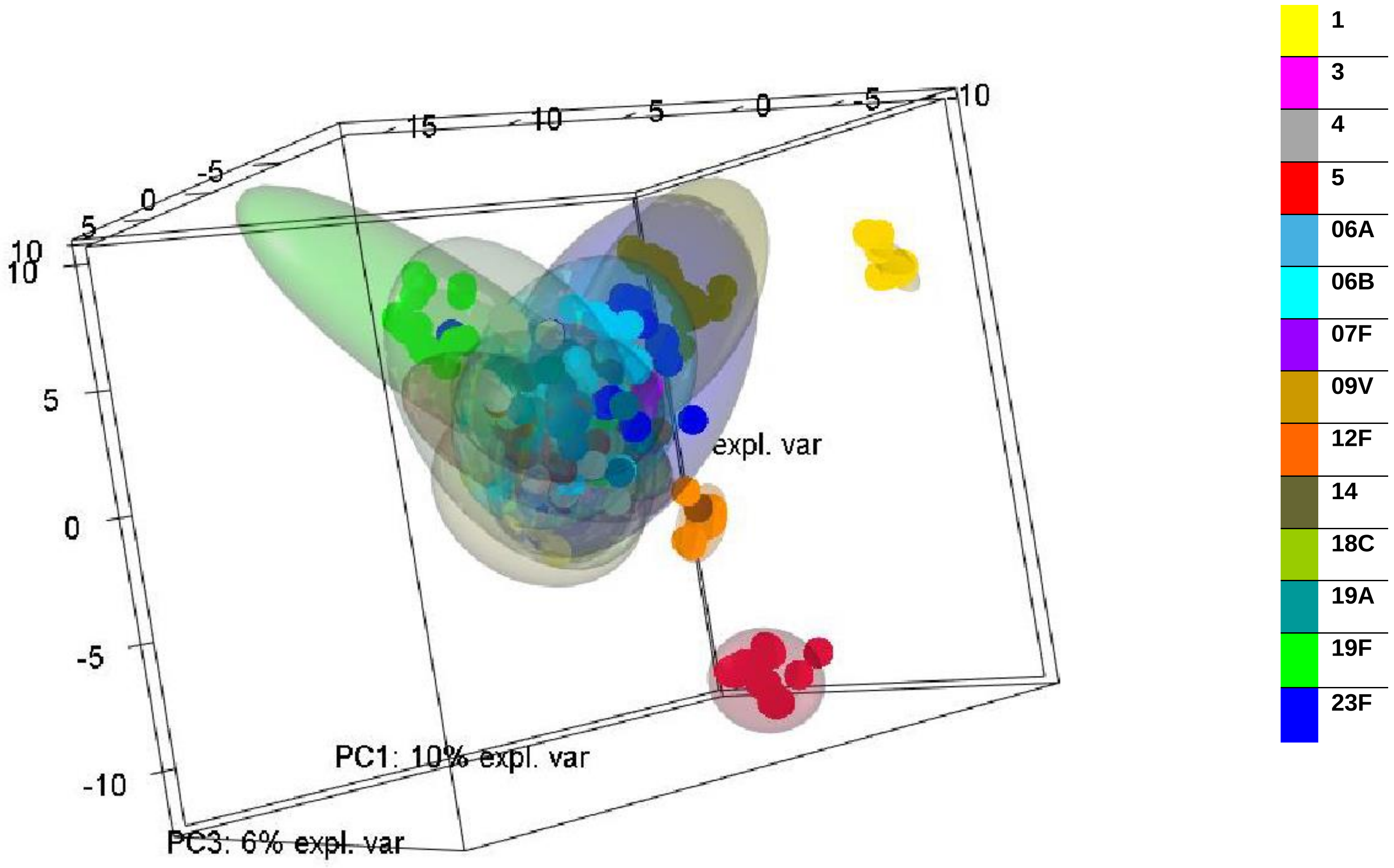

S9 Fig. Principal Component Analysis (PCA) of gene distribution for down sampled hyper-invasive serotypes (1, 5, and 12F) and those included in the Pneumococcal Conjugate Vaccine 13 (3, 4, 06A, 06B, 07F, 09V, 14, 18C, 19A, 19F, and 23F). For each serotype, 10 isolates were randomly selected from the nasopharynx, blood, and cerebrospinal fluid. For serotype 1, only seven isolates from nasopharynx were available. Hyper-invasive serotypes 1, 5, and 12F exhibited distinct clustering compared to other serotypes.
